# *Rosa rubiginosa L*. Extract Rich in Bioactive Phenolics Possess Potent Anti‐Inflammatory, Antioxidant, Photoprotective, and Antimicrobial Biofunctional Activities

**DOI:** 10.1002/cbdv.71418

**Published:** 2026-06-15

**Authors:** Sophia Letsiou, Konstantina Kyratzi, Aliki Tsakni, Dimitrios Kranas, Konstantinos Pavlidis, Anna Ofrydopoulou, Panagiotis Halvatsiotis, Dimitra Houhoula, Alexandros Tsoupras

**Affiliations:** ^1^ Department of Food Science and Technology University of West Attica Athens Greece; ^2^ Hephaestus Laboratory School of Chemistry Faculty of Sciences Democritus University of Thrace Kavala Greece; ^3^ 2nd Propaedeutic Department of Internal Medicine Medical School “ATTIKON” University Hospital National and Kapodistrian University of Athens Chaidari Greece

**Keywords:** anti‐inflammatory, antimicrobial, antioxidant, carotenoids, PAF inhibition, PAF receptor, photoprotective, *Rosa rubiginosa L*

## Abstract

The hydroethanolic *Rosa rubiginosa* extract is evaluated in vitro for antioxidant, anti‐inflammatory, antiplatelet, photoprotective, and antimicrobial activities. Rich in phenolics and carotenoids, it shows strong antioxidant activity (notably in DPPH) and high SPF‐related UV protection. Major compounds include rosmarinic acid, hydroxybenzoic acid, quercetin, and luteolin. Synergistic interactions among bioactives enhance antioxidant capacity beyond that of individual compounds. The extract demonstrates pronounced anti‐inflammatory effects against platelet‐activating factor (PAF), exceeding those of isolated standards, while showing lower activity against ADP‐induced platelet aggregation, indicating specificity toward PAF‐mediated pathways. Molecular docking supports interactions with the PAF receptor. Given PAF's role in UV‐induced inflammation and melanoma, the extract's UV absorption, antioxidant capacity, and anti‐PAF activity suggest protective effects against photoaging. Antimicrobial activity is stronger against Gram‐negative bacteria, with slower effects on Gram‐positive strains. Overall, *R. rubiginosa* extract exhibits broad bioactivity, supporting its potential as a multifunctional ingredient in functional foods, nutraceuticals, supplements, and cosmetic formulations.

## Graphical abstract

A rich in bioactive phenolics extract from *Rosa rubiginosa L*. exhibits strong antioxidant, photoprotective, anti‐inflammatory, and antimicrobial activities. Synergistic interactions among bioactives enhance efficacy, particularly against PAF‐mediated pathways. These findings highlight its potential as a multifunctional ingredient for nutraceutical, cosmetic, and anti‐photoaging applications, inviting further exploration of its therapeutic value.

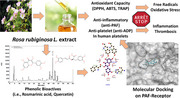

## 1 Introduction

In recent years, edible flower extracts have emerged as promising functional ingredients in the fields of nutrition and health sciences due to their rich phytochemical content and diverse biological properties. These natural products are increasingly recognized not only for their aesthetic and culinary appeal but also for their potential to mitigate oxidative stress, inflammation, and chronic disease risk. Notably, anthocyanin‐rich extracts from flowers such as butterfly pea (*Clitoria ternatea*) have demonstrated significant antioxidant capacity, effectively scavenging free radicals, protecting lipid membranes, and reducing lipid peroxidation both in vitro and in vivo [1].

Among the most extensively studied genera is Rosa, belonging to the Rosaceae family, which comprises approximately 200 species, most of which are deciduous shrubs [2, 3]. Rose species are traditionally highly valued for their nutritional, medicinal, ornamental, and cosmetic applications. Their fruits, commonly referred to as rose hips, are widely used in teas, jams, jellies, and dietary supplements due to their high content of vitamins, flavonoids, and phenolic acids [2, 3]. Numerous species within the genus have shown a wide spectrum of pharmacological activities, including antioxidant, anti‐inflammatory, antidiabetic, hepatoprotective, antimicrobial, anticancer, anti‐arthritic, neuroprotective, and anti‐obesity effects [2, 3]. In addition, several Rosa species have already demonstrated molecular mechanisms of bioactivity. Specifically, it has been reported that *Rosa rugosa* petal extract inhibited iNOS and COX‑2 expression, lowered NO and PGE_2_ production, and downregulated NF‑κB/MAPK signaling in LPS‐activated macrophages [3, 4]. Similarly, hydroalcoholic petal extracts and essential oils from *Rosa damascena* showed strong DPPH radical scavenging, lipid peroxidation inhibition, and reduced oxidative stress in endothelial models PMC [3, 5], while fruit extracts from *Rosa canina* (rose hips) displayed high phenolic content and in vivo antioxidant and anti‐inflammatory effects—decreasing cytokines, COX‑2/iNOS activity, NF‑κB signaling, and improving osteoarthritis outcomes [3, 6]. The other hand, *Rosa rubiginosa L*. (sweet briar rose), a wild rose species native to Europe and Western Asia, is primarily recognized for its high content of polyunsaturated fatty acids and antioxidant phenolics in its fruit and seeds [7, 8]. While its antioxidant potential is well‐documented in vitro, there is a paucity of research exploring its anti‐inflammatory capacity, particularly in relation to platelet‐activating factor (PAF)—a potent lipid mediator implicated in inflammation and thrombosis, and associated chronic disorders, including cardiovascular disease and cancer [9, 10]. Inhibition of PAF signaling represents a novel anti‐inflammatory strategy beyond traditional cytokine modulation and COX inhibition [9, 10, 11, 12, 13].

To address this knowledge gap, we hypothesize that *R. rubiginosa* extracts exhibit multi‐target bioactivity, including antioxidant, anti‐inflammatory, and anti‐PAF effects. Therefore, the aim of this study is to provide the first integrated in vitro assessment of *R. rubiginosa* extract with respect to: (1) its inhibitory effect on PAF‐induced platelet aggregation; (2) its anti‐inflammatory potential via phytochemical profiling and functional assays; and (3) its antioxidant and photoprotective capacity using multiple bioassays. Moreover, molecular docking was also performed to investigate the binding capacity of the most bioactive phytochemicals on PAF‐receptor, providing a structure activity relationship for these natural phenolic bioactives. By uncovering these combined bioactivities, we aim to position *R. rubiginos L*. as a versatile and sustainable biofunctional ingredient with applications in functional foods, nutraceuticals, and nutricosmetics.

## 2 Results and Discussion

In this study we investigated the *R. rubiginosa* extract in vitro properties to shed light on its use as biofunctional ingredient in foods, nutraceutical, nutricosmetics or cosmeceuticals. Thus, we focused on anti‐inflammatory, antioxidant as well as antimicrobial properties of the hydroalcoholic extract from this plant. Plants were commercially purchased and identified at the University of West Attica. The yield of extraction was 95%, according to the methodology applied by Letsiou et al. [14].

### 2.1 Total Carotenoid Content (TCC) of the *R. rubiginosa* Extract

The analysis of the *R. rubiginosa* extract for the determination of the TCC exhibited a high carotenoid concentration, approximately of 55.45 mg CE per g of dry weight (DW), indicating a remarkably high content of these lipophilic phytochemicals. Generally, carotenoids are a class of naturally pigments responsible for distinctive hues in many plant tissues. Structurally, carotenoids often consist of conjugated double bonds, which grant significant antioxidant activity. The antioxidant mechanism involves quenching singlet oxygen and scavenging peroxyl radicals, thereby protecting cells from oxidative stress, which can be caused by both exogenous and endogenous factors. Carotenoids like β‐carotene and lutein are known for taking part in the stabilization of biological membranes as well as in the reduction of lipid peroxidation, with strong antioxidant and anti‐inflammatory protection that promote anti‐aging and photoprotective benefits for skin health [15].

In comparison to other types of roses such as cultivated roses (*R. gallica*), the sample that was examined in this study showed higher content of carotenoid bioactive compounds than the cultivated ones, which can be explained by the high concentration of the extract. Additionally, a study on *Calendula officinalis* measured a concentration of carotenoids about 35 mg/g DW, while a study on *Tagetes erecta* reported a carotenoid content between 2.6–4.3 mg/g DW [16, 17]. Thus, the rose extract in this study exceeded both the other flowers, suggesting it is richer than most studied edible flowers. It is important to mention that the examined hydroethanolic solution that was used and evaporated to isolate the lipid extract, contained 0.1 mg of rose extract per 1 mL of solution.

### 2.2 Antioxidant Activities

Τhe *R. rubiginosa* extract has shown previously potent antioxidant capacity detected via the DPPH assay [14]. In the present study, we evaluated with the same assay again the antioxidant capacity of this extract but this time in comparison to a very potent antioxidant standard compound, Trolox, which is a synthetic analogue of vitamin E, in order to normalize our results, which facilitated comparisons with other similar extracts assessed in the same conditions. It was found that the *Rose* L. extract exhibited significant radical scavenging activity with an IC_50_ comparable to that of Trolox when assessed at the same conditions of the DPPH assay, providing an absolute value of the Trolox Equivalent Antioxidant Capacity (TEAC) of 3.96, which is higher than that of 1 indicating that the extract belongs to the most potent antioxidant bioactives as it is comparable to that of Trolox. The highest the TEAC value for a compound the highest its DPPH‐based antioxidant bioactivity. As shown in Table 1 the TEAC value of the extract is much higher than the TEAC values of several control phenolics that were assessed in the same conditions. This result indicate a synergism that seems to take place within the extract due to the copresence of more than one of such compounds present in this extract. Interestingly, similar synergistic effect was also observed to a food supplement containing extract of flavonoids from Rosehips and Citrus fruits, both on their own and in the presence of vitamin C, which also showed similar TEAC values with the ones observed in the present study for the *R. rubiginosa L*. extract [18].

**TABLE 1:** Antioxidant capacity of the *Rosa rubiginosa L*. extract compared to standard phenolics and other similar extracts used in supplements, assessed in the same conditions, of the DPPH, ABTS, and FRAP assays.

Assay	DPPH (TEAC value^a^)	ABTS (ABTS value^a^)	FRAP (FRAP value^a^)	
Sample	Range	Range	Range	Reference
*Rosa rubiginosa L*. extract	2.83–6.48	9.72–14.56	16.23–20.31	—
P coumaric acid	0.0028–0.0041	5.9–13.7	ND	—
Rosmarinic acid	0.0059–0.0071	17.1–26.0	39.1–49.5	—
Rutin	0,0149–0.0197	11.8–43.7	101.4–105.0	—
Quercetin	0.00043–0.00409	19 813.34–70 290.18	2149.81–2398.79	[18]
Catechin	0.00536–0.00550	13.92–14.08	2428–2468	[18]
Curcumin	0.00077–0.00171	700.67–1366.47	147.01–248.65	[18]
Vitamin C	2.7–4.0	7485.8–589 051.4	1331.75–6944.03	[18]
Extract of flavonoids from rosehips and citrus fruits	5.4–8.02	14 971.5–1 178 102.9	2663.51–13 888.05	[18]

^a^ Expressed either as TEAC values, (TEAC = IC_50_ of Trolox/IC_50_ of sample assessed within the same conditions, according to the DPPH assay), as ABTS values (µmol of Trolox equivalent (TE)/g DW of sample, according to the ABTS assay) and as FRAP values (µmol of TE/g DW of sample, according to the FRAP assay). Abbreviations: DW = dry weight.

The DPPH is a widely used method for evaluating the antioxidant activity based on the ability of compounds to donate hydrogen atoms or electrons to the stable free radical DPPH. Thus, it is an assay that primarily reflects hydrogen atom transfer (HAT) mechanisms, favoring compounds capable of donating hydrogen atoms, such as phenolic antioxidants typically being present in amphiphilic fractions or hydroalcoholic extracts like the present one. The strong antioxidant activity of the rose extract in this assay suggests a high concentration of hydrogen‐donating atoms, such as phenolics and flavonoids, which are known to be found in flowers. Carotenoids may also have contributed to the strong antioxidant capacity, although their role in the DPPH scavenging is less direct compared to phenolic compounds. The results of this assay indicated a high potency of this extract for further functional food or cosmetic applications, as previously observed for a flavonoids‐rich extract from rosehips and citrus fruits with similar TEAC values, which is used in supplements with vitamin C [18].

The antioxidant activity of the rose extracts was also evaluated using the ABTS assay. The ABTS assay is based primarily on single electron transfer (SET) reactions, which can involve a broader range of antioxidant molecules, including lipophilic compounds such as tocopherols and carotenoids. More specifically, in this assay. It involves the creation of the ABTS^+^• cation radical, which is highly reactive and is reduced in the presence of hydrogen‐donating antioxidants. This reaction is typically broader than DPPH, because it can detect both hydrophilic and lipophilic antioxidant compounds [19, 20]. Regarding the rose extract, the ABTS assay further confirmed its antioxidant capability, with an ABTS value of 11.84 µmol of Trolox equivalent (TE)/g DW. The high ABTS scavenging capacity of the rose extract indicates the presence of multiple antioxidant compounds, which can act either via SET or HAT mechanisms [19]. The ABTS value reflects strong radical quenching ability, suggesting a synergistic effect between various phytochemicals, including polyphenols, carotenoids, and ascorbic acid derivatives [21]. Based on previous reports, the ABTS value of this extract was lower than some highly potent floral extracts and standard phenolics and a supplement containing Rosehips and citrus flavonoids extracts [18] (Table 1). For example, pink Prunus domestica flowers exhibited significantly higher ABTS activity, with values reaching 65 µmol TE/g DW, attributed to their intense pigmentation and high polyphenolic content [22]. Moreover, differently than what was observed in the DPPH assay, in the ABTS assay the extract showed lower activity than that of several phenolic standards or even from that observed in a Rose extract used in a supplement with vitamin C (Table 1), indicating that the phenolics of the extract cannot act synergistically on SET, concluding in a relative low ABTS value for the extract.

In the FRAP assay, the rose extract demonstrated a FRAP value of 18.48 µmol TE/g DW, reflecting that the extract possess some ability of reducing power. The FRAP assay measures the antioxidant ability to reduce ferric iron to ferrous iron. Unlike DPPH or ABTS, FRAP primarily assesses electron donating capacity and it does not respond to compounds that act solely via HAT. Therefore, it can be considered as a good indicator of overall reducing potential rather than radical scavenging alone, and thus it is proposed as the closest to in vivo conditions antioxidant assay, as it imitates the reducing power for iron in blood plasma. Compared to other types of flowers, the rose extract that was examined in this study, in terms of the FRAP assay, aligns with the upper range of values reported for rose petals (11.5–20.9 µmol TE/g DW) [23, 24]. In another study, which investigated 23 types of flowers the FRAP activity ranged from 8.08 µmol TE/g DW, up to 913.6 µmol TE/g DW, illustrating the broad spectrum of antioxidant capacities in such floral extracts. More specifically the rose extract that was examined in this study, exceeded 4 types of flowers in the above‐mentioned study [25]. However, in this assay too the extract showed lower activity than that of several phenolic standards or even from that observed in a Rosehips extract with citrus fruits flavonoids used in a supplement with vitamin C [18] (Table 1), indicating that the phenolics of the extract did not act synergistically on reducing the Ferric Power, concluding in a relative low FRAP value for the extract.

In general, all the antioxidant results and especially those from the DPPH assay align well with findings from Chilean *R. rubiginosa* fruits, where DPPH activity reached approximately 122–125 µmol TE/g FW and CUPRAC–TEAC values correlated with high flavonol/catechin content, particularly in samples from specific localities [26], as well as with the results of the DPPH‐based TEAC values of the Rosehips extract with citrus fruits flavonoids used in a supplement with vitamin C [18] (Table 1) (Chrysikopoulou et al.). Similarly, Sicilian specimens of *R. rubiginosa* exhibited among the highest vitamin C and carotenoid concentrations compared to other Rosa species, corroborating our extract's elevated carotenoid and phenolic levels [27]. These comparative studies reinforce that *R. rubiginosa* fruits are among the richest Rosa species in these functional phytochemicals.

### 2.3 Anti‐UV Photoprotective Effects of the *R. rubiginosa* Extract

**FIGURE 1:**
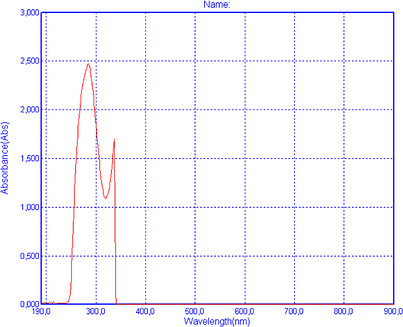
UV–vis spectra of *R. rubiginosa* extract.

Beyond its antioxidant properties, the *R. rubiginosa* extract was evaluated for its UV–vis absorption profile. As shown in Figure 1, the extract exhibited distinct absorption peaks within the UVA (320–400 nm) and UVB (280–320 nm) regions. Moreover, sun protection factor (SPF) of the extract was found to be within the range of 15–20 (min 15.0, median 16.1, max 19.7), calculated via the use of UV–vis spectroscopy, recording the absorption of the samples at wavelengths from 290 to 320 nm, every 5 nm, as previously described by Tsiapali et al. [28]. The photoprotective action of a compound for sunscreen is measured universally by the SPF which establishes the increase of the dose of sun exposure with the photoprotective product applied without the occurrence of erythema. These values of SPF (15–20) for the extract provide a strong indication for photoprotection by the presence of compounds capable of absorbing UV radiation, likely associated with phenolic constituents such as flavonoids and related phytochemicals.

However, it should be noted that UV–vis absorption and SPF calculation alone does not directly demonstrate biological photoprotective or anti‐aging effects. Therefore, these results should be interpreted as indicative of UV–absorbing potential, rather than confirmed functional activity. Further studies, including cell‐based and in vivo assays, are required to validate any photoprotective or antiaging efficacy.

### 2.4 Anti‐Inflammatory and Antiplatelet Properties of *R. rubiginosa* Extract

**FIGURE 2:**
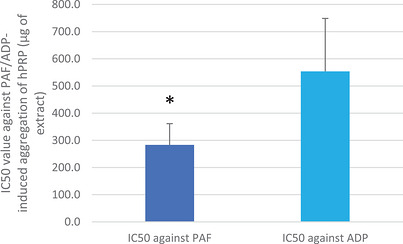
Anti‐inflammatory (Anti‐PAF) and antiplatelet (Anti‐ADP) effects of *R. rubiginosa* extract. Results are expressed as IC_50_ Values (half maximum inhibitory activity) against the aggregation of hPRP induced by the thrombo‐inflammatory PAF‐associated pathways or by the standard‐classic platelet agonist, ADP. *Indicates a statistically significant difference (*p* < 0.05) based on a one way ANOVA compare means statistical methodology, for the anti‐PAF effects of the extract in comparison to its anti‐ADP effects (The lower the IC_50_ value for a thrombo‐inflammatory mediator the higher the inhibittory activity of the extract against this mediator). (PAF, platelet‐activating factor; hPRP, human plasma rich platelets).

PAF is a potent inflammatory mediator implicated in several inflammation related pathological situations, including photoaging, melanoma, other types of cancer and neurodegenerative and cardiovascular disorders [9, 10, 11, 12, 13]. PAF exerts its distinct effects by binding on a specific G‐protein coupled membrane receptor (PAFR) that is present in all cells of our body, including platelets. Thus, an effective cell‐model that allows the quantification of the anti‐inflammatory anti‐PAF inhibitory effects of a bioactive compound/extract/drug against the PAF‐related pathway is by assessing the ability of this bioactive to inhibit PAF‐induced aggregation of platelets in the presence of a range of concentrations for this bioactive, from which the half maximum inhibitory activity (IC_50_ value) for this bioactive can be obtained via well‐established methodology, either for drugs or for natural extracts [13, 29]. The IC_50_ for a bioactive extract against the PAF pathway in platelets provides a strength of its inhibitory effect against his inflammatory mediator, since the lower the IC_50_ value for an extract the stronger its anti‐PAF activities. In the present study the anti‐PAF anti‐inflammatory effects of the Rose extracts were evaluated in human platelet rich plasma (PRP) by quantifying their IC_50_ values against PAF‐induced aggregation of PRP, in comparison to their effects to a standard platelet agonist, ADP, which also induce platelet aggregation but through a different thrombotic pathway. Results are shown in Figure 2.

As shown in Figure 2, the anti‐PAF inhibitory effects of the rose extracts ce were found to be statistically significantly stronger than those of the extract against the ADP pathway, suggesting that the extracts show some specificity against the inflammatory pathway of PAF rather than the thrombotic pathway of ADP. In other words, the extract seems to contain more potent anti‐inflammatory anti‐PAF‐inhibitors than antiplatelet anti‐ADP‐inhibitors. Taking into account that PAF is strongly associated with photoaging induced melanoma, then combining these results for strong anti‐PAF activities of this extract, along with its high photoprotective SPF values further suggest a promising strong protective efficacy against photoaging and melanoma for this extract.

The inhibition of the ADP pathway is primarily linked to classic thrombosis prevention and antiplatelet therapies, while the stronger inhibitory effect on the PAF pathway indicates that this extract may have higher specificity and be more effective in suppressing inflammatory responses not only in platelets but in other tissues and organs too that are affected by the PAF pathway as they possess PAF‐receptor. Nevertheless, taking also into account that platelet activation and aggregation induced by such thrombo‐inflammatory mediators are usually implicated in cardiovascular disorders and cancer [8, 9], then the combined anti‐inflammatory and antithrombotic capacity of this extract that was observed in the present study in human platelets further suggest an overall cardioprotective efficacy and antitumor potency for this extract. However, in vivo outcomes from a targeted clinical trial based on a nutraceutical containing such an extract are needed to fully support such a statement.

Our study adds novel evidence of selective anti‐PAF platelet aggregation inhibition (IC_50_ ≈ 34.5 µg/mL), a mechanism not widely reported in prior rosehip literature for a rosehip extract on its own, apart from a study on rosehip extract of flavonoids in the copresence of flavonoids from citrus fruits and vitamin C, in which better anti‐PAF effects were observed (lower IC_50_ value) due to synergism between all flavonoids and vitamin C, as explained at the specific article [18]. In contrast, rosebud extracts from hybrid roses (“Pretty Velvet”, “Hanggina”) inhibited NO and PGE_2_ production in LPS‑activated macrophages, via COX‑2 and iNOS suppression—mechanisms more related to classical inflammation pathways [30]. While both polyphenols and terpenes are implicated, our results further suggest *R. rubiginosa* may also interfere with lipid‑mediator pathways such as PAF, offering complementary anti‐inflammatory activity. Overall, the findings of the present study also support the use of the rose extracts as sustainable and cost‐effective sources of functional bioactives, suitable for valorisation in food, cosmetic, or supplement and nutraceutical formulations.

Moreover, a standard solution of the main component, rosmarinic acid (RA), and the less component of the extract, p‐coumaric acid, were also assessed in human platelets and the results of their anti‐PAF anti‐inflammatory activity and their anti‐ADP antiplatelet capacity, expressed as IC_50_ values (µM of the phenolic bioactive compound that can achieve half maximum inhibition of PAF/ADP‐induced platelet aggregation) that are shown in Table 2. In the same table previously published outcomes for the IC_50_ against these two pathways of platelet aggregation of specific well‐established anti‐PAF and anti‐ADP inhibitors and drugs are also presented (i.e., IC_50_ values for Phosphatidylcholine bearing esterified the omega‐3 fatty acid, docosahexaenoic acid (DHA) in its glycerol backbone, Ginkgolide B, Aspirin and Clopidogrel), as well as the IC_50_ values of quercetin, another highly bioactive ingredient present in this extract, and of other bioactive flavonoids (i.e., rutin, catechin) are also presented to facilitate comparisons

**TABLE 2:** IC_50_ values (in µM) of rosmarinic acid and of other highly bioactive phenolic compounds and well established anti‐PAF and anti‐ADP inhibitors against PAF/ADP‐induced platelet aggregation (*N* = at least 6–12 blood samples from different donors).

Bioactive compound	IC_50_ against PAF^a^	IC_50_ against ADP^a^	Reference
Rosmarinic acid	140–284	335–566	—
p‐Coumaric acid	468–667	686–906	—
rutin	51–119	291–493	—
Quercetin	205–850	100–200	[18]
Catechin	180–400	2000–6000	[18]
Curcumin	150–250	90–170	[18]
Phosphatidylcholine bearing esterified omega‐3 fatty acids in its glycerol backbone	1.2–1.8	2.1–2.5	[29, 31]
Ginkgolide B	35–100	ND	[31]
Aspirin	180–400	10–110	[12, 31]
Clopidogrel	130–450	1400–4500	[12]

^a^ Expressed as a range (min–max) of µM of the natural bioactive compound or drug that can achieve half maximum inhibition (IC_50_) of PAF/ADP‐induced platelet aggregation

The results showed that RA exhibited very strong anti‐PAF activity and lower anti‐ADP activity in human platelets, suggesting a higher specificity for the inhibition of this natural phenolic bioactive against the PAF‐related thrombo‐inflammatory signaling of platelet aggregation, while quercetin has previously shown stronger inhibitory effects against both PAF and ADP too, in contrast to the higher anti‐PAF specificity that was observed for RA or other flavonoids like rutin and catechin. On the other hand, p‐coumaric acid showed lower but considerable anti‐PAF and anti‐ADP activities compared to those observed in RA and quercetin, without any specificity against any of the two pathways, suggesting a general antiplatelet effect for this natural simple phenolic bioactive. Moreover, the anti‐PAF activities of both RA and quercetin were of the same level of magnitude and thus comparable to the anti‐PAF activities previously observed for other highly bioactive phenolics, including flavonoids like rutin and catechin that also exhibit higher specificity against PAF [18] and polyphenols like curcumin [18], as well as comparable to the anti‐PAF and anti‐ADP effects of well‐established specific anti‐PAF and antiplatelet inhibitors like natural derived phospholipids bearing omega‐3 fatty acids [29, 31] ginkgolide B [31], and drugs like aspirin [12, 31] and clopidogrel [12].

### 2.5 Phytochemical Characterization of *R. rubiginosa* Extract Using HPLC

**FIGURE 3:**
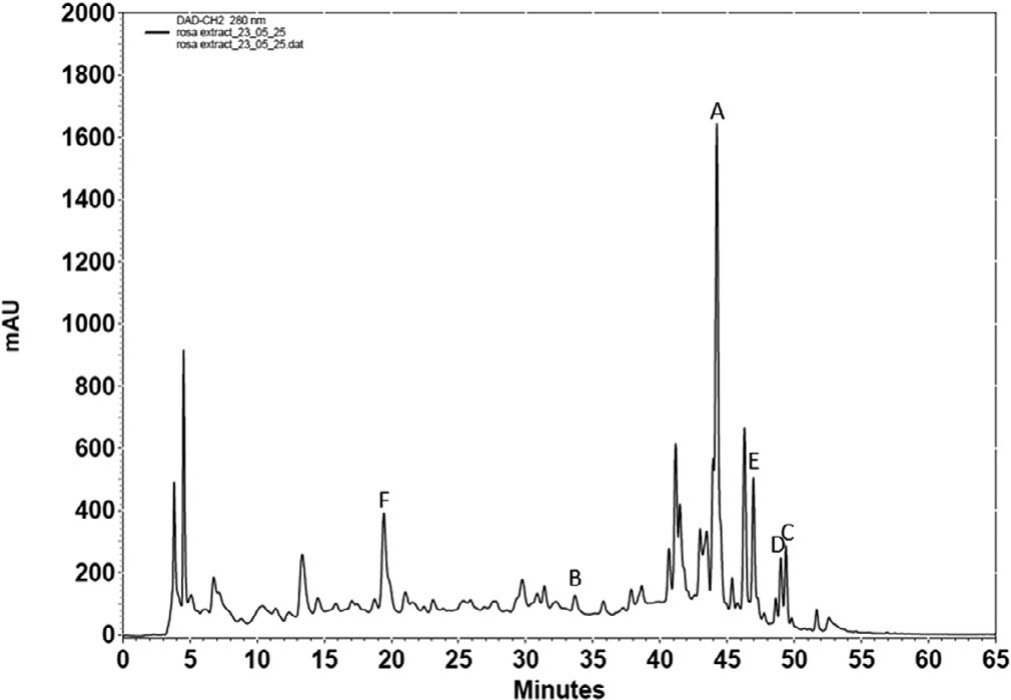
Phytochemical analysis (HPLC‑DAD) of *R. rubiginosa* extract.

Several bioactive phenolic compounds were found in the *R. rubiginosa* extract at varying levels through high performance liquid chromatography analysis (Table 3, Figure 3). Typical calibration curves and representative chromatograms for each one of the main phenolic bioactives identified in the extract can be seen at the Figures S1–S11 and Table S1. Based on this analysis, RA was found to be the most prevalent compound (376.86 µg/mL). This polyphenol is recognized for its antioxidant, anti‐inflammatory, neuroprotective, and antibacterial properties [32, 33]. Its high concentration indicates that it contributes significantly to the extract's total bioactivity, potentially increasing its therapeutic effectiveness.

**TABLE 3:** Concentration (in µg/mL) of 6 tested metabolites included in the extract of *Rosa rubiginosa L*.

Bioactive compound	Concentration (µg/mL)
Α. Rosmarinic acid	376.86
Β. Coumaric acid	29.02
C. Luteolin	81.30
D. Quercetin	69.94
E. Eriodictyol	66.82
F. Hydroxybenzoic acid	296.28

Hydroxybenzoic acid (HΒA) was the second most abundant compound (296.28 µg/mL), reflecting its known widespread presence in plant tissues as an antioxidant [34]. Flavonoids like luteolin (81.30 µg/mL), quercetin (69.94 µg/mL), and eriodictyol (66.82 µg/mL) were present in appreciable amounts, and are recognized for their diverse biological activities, including anti‐inflammatory, antiviral, and neuroprotective properties [32], while they seem to interact with the binding of PAF on its receptor (PAF‐Receptor) based on binding experiments with ^3^H‐PAF [35]. Moreover, quercetin exhibits strong radical scavenging properties and modulate signaling pathways in cells [36].

Despite being present at a lower concentration (29.02 µg/mL), coumaric acid (CA) is also important to the extract's antioxidant profile and has been associated with cardiovascular and antimicrobial advantages as well [37].

The wide range and high concentration of phenolic components in *R. rubiginosa* extract support its traditional use in herbal medicine and highlight its potential for future pharmacological investigation. The findings suggest that *R. rubiginosa* may be a rich source of bioactive compounds with synergistic antioxidant capabilities, which further supports its potential use in functional foods or nutraceutical formulations.

Our HPLC‑DAD analysis highlighted RA, quercetin and luteolin as predominant phenolics. In Sicilian rosehip comparisons, R. canina displayed higher total phenolic content overall, but *R. rubiginosa* excelled in vitamin C and carotenoids [27]. In addition, the same study revealed that the integrity of Caco‑2 epithelial monolayers was affected at high extract concentrations for *R. rubiginosa* and *R. corymbifera*, whereas *R. canina* did not alter TEER values significantly [27]. This suggests that while *R. rubiginosa* is bioactive, its gastrointestinal tolerance warrants further dose‐ranging investigations [38].

With respect to the anti‐inflammatory efficacy of the extract, these results come to compliment the anti‐PAF ones observed for the examined extract. More specifically, the contribution of RA alone may not fully explain the observed IC_50_ value of the whole extract. The concentration of RA in the extract (376.86 µg/mL in the stock solution) corresponds to a lower effective concentration at the IC_50_ of the extract, which may not fully account for the observed anti‐PAF activity when considered alone. It should be emphasized at this point that the anti‐PAF efficacy of such extracts does not usually depend in the presence of only one compound, rather than in the copresence of such compounds that usually tend to act synergistically. This was also observed in a similar study recently described by Chrysikopoulou et al. [18], in which the copresence of some flavonoids (catechin and quercetin) did not just provided a total additive of their anti‐PAF potency, rather than provided a multiplied activity with at least 10 times higher efficacy, as an order of magnitude lower concentration of both flavonoids were needed when assessed together against PAF in the platelet aggregometry assay, suggesting a strong synergism against this inflammatory pathway. Similar synergism of flavonoids was also observed in the same study for a supplement containing flavonoids from rosehips and citrus fruits, along with vitamin C [18]. Moreover, the copresence of other bioactive amphiphilic compounds that are usually present in such extract, like carotenoids and polar lipids can also contribute in a more complex synergistic effect with the bioactive phenolics and thus provide a much more potent anti‐PAF anti‐inflammatory efficacy for the whole extract versus each bioactive compound when assessed on its own.

### 2.6 Molecular Docking of Bioactive Phenolics for PAF‐Receptor

#### 2.6.1 Docking of RA With PAFR

PAFR (platelet‐activating factor receptor) is a receptor coupled to a G protein that is linked to PAF (“Platelet‐activating factor (PAF) receptor and genetically engineered PAF receptor mutant mice—PubMed,” n.d [39].). Clinical and preclinical studies highlight PAF and PAFR detrimental role: play an important pathophysiological role in mediating inflammatory and oxidative reactions, such as bronchial asthma, endotoxin shock, central nervous system disorders, and skin diseases, activating downstream signaling cascades involved in them [8, 9, 10, 40]. Several phenolic bioactives and especially flavonoids from plant extracts like those being present in the *R. Lubiginosa L*. extract have shown stron g anti‐PAF activities, while they have also been evaluated for their effect on PAF‐receptor binding on rabbit platelets using ^3^H‐PAF as a ligand [35]. The majority of the flavonoids have previously exhibited > 50% inhibition on the binding activity of PAF at 18.2 microg/ml. These flavonoids were identified as methyl ethers of eriodictyol, quercetin, naringenin, kaempferol, taxifolin, with IC_50_ values ranging from 19.5 to 62.1 microM [35]. However, this methodology has the limitation that it needs to produce methyl ethers of such flavonoids to interact with the PAF‐recpetor, and thus it has not yet been able to show the binding of how the original flavonoid molecules themselves bind to PAF‐R and how they potentially can block the binding of PAF to PAF‐Receptor, according to their observed strong anti‐PAF capacity (low IC_50_ values against PAF‐induced platelet aggregation). Thus, more adept and modern state‐of‐the‐art techniques are needed to do so, like the molecular docking in silico assessment performed in the present study.

With respect to the active phenolic and flavonoid substances that are present in the *R. Lubiginosa L*. extract and have shown strong anti‐PAF effects, RA is a water‐soluble phenolic compound with various biological activities, including antioxidant, anticancer, and anti‐inflammatory properties [33]. Research has also studied the antiplatelet property of RA, demonstrating that it causes a remarkable inhibition of platelet aggregation, which was caused by the use of repellents such as arachidonic acid (AA), ADP, and collagen, providing promising inhibition of ERp57, a receptor associated with platelet aggregation [41], also observed in the present study for ADP and PAF too.

**FIGURE 4:**
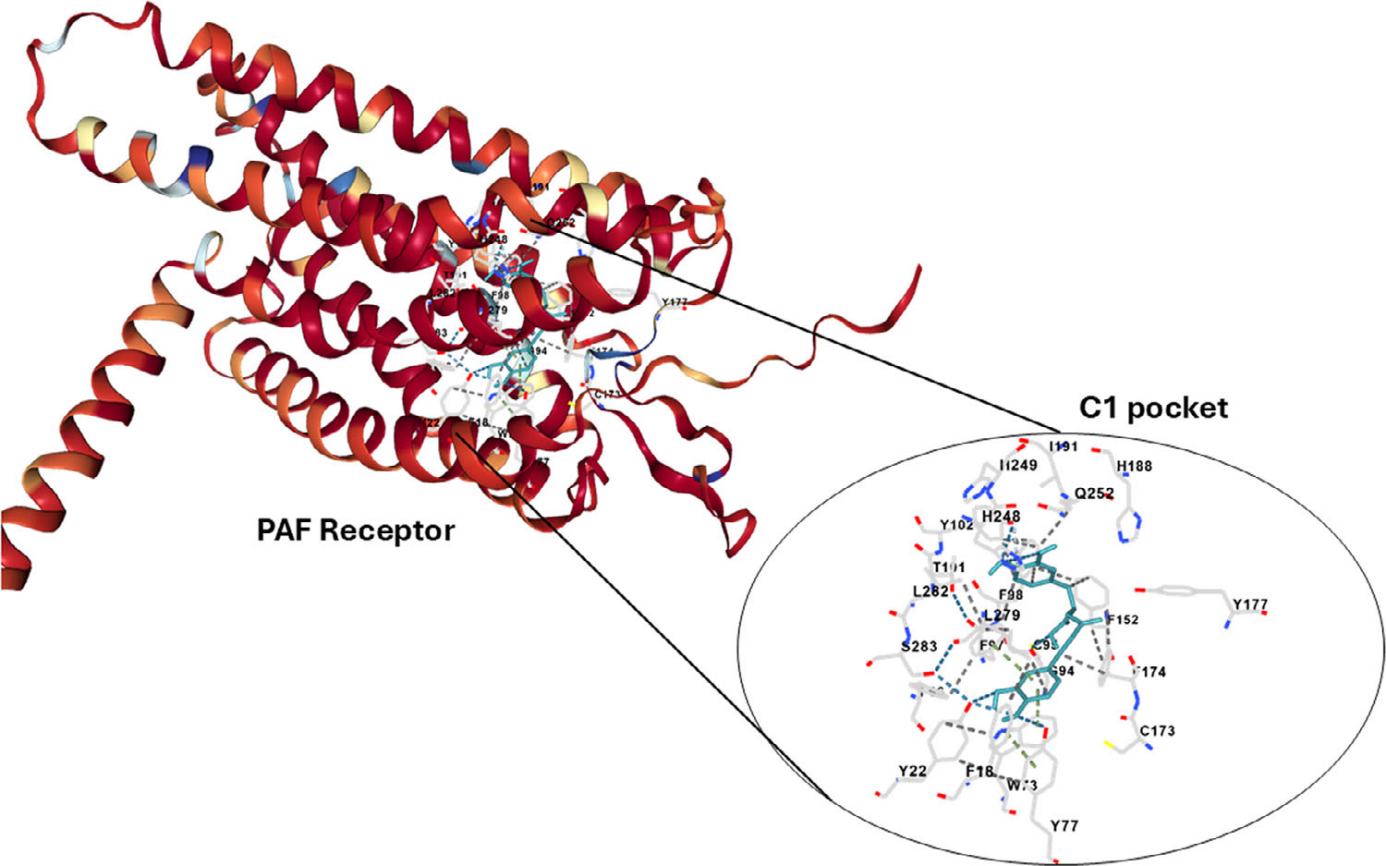
Active pocket C1 of PAF receptor to which all selected compounds were bound. The magnification shows the placement of the Rosmarinic acid molecule in the C1 cavity and which amino acids it interacts with when placed in it. This position yielded the optimal binding scores for the compounds, and selected for the docking analysis.

**FIGURE 5:**
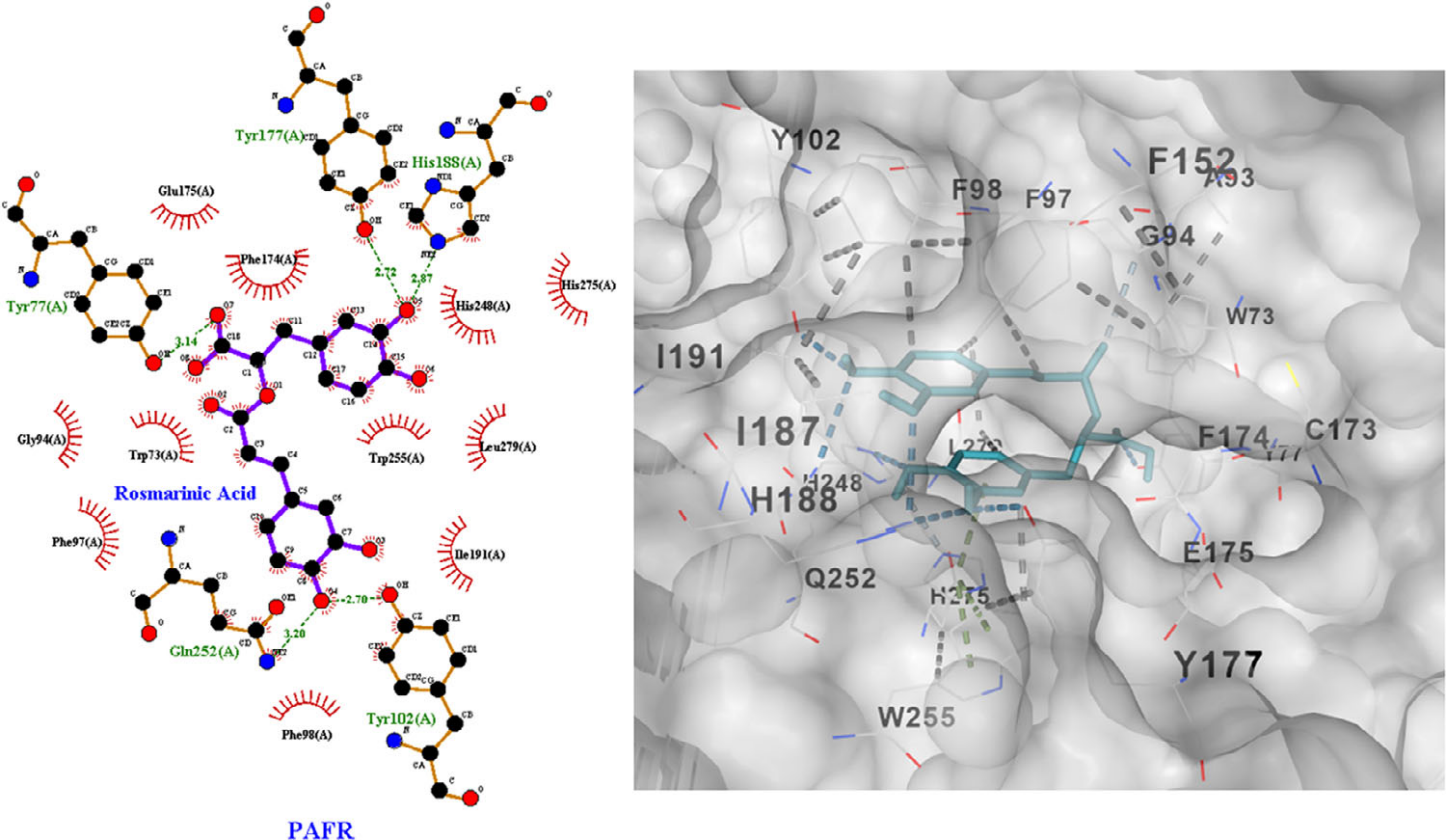
Two‐dimensional interaction diagram (left) and 3D binding pose (right) of rosmarinic acid (RA) docked to the PAF receptor. Hydrogen bonds between RA and PAFR highlighted in the 2D diagram and residues Tyr77, Tyr102(A), Tyr177(A), His188(A), and Gln252(A) (green labels), as well as hydrophobic interactions with Phe98(A) and Phe174(A) (red arcs). The positioning of RA within the PAFR binding pocket and the resulting key interactions with amino acid residues are shown in the 3D structure.

RA demonstrated a favorable and strong connection to PAFR, as indicated by the binding score of −8.6 kcal/mol according to blind docking in AutoDock Vina (Table 4), by in silico molecular docking analysis, which was performed as previously described by Tsiapali et al. [28]. The active compound appears to be located in the binding pocket C1 of PAFR (Figure 4), with a cavity volume (6073 Å^3^), occupying a large volume of the cavity and forming both polar and nonpolar interactions (Figure 5). These interactions, which contribute to the stability of the binding complex and are also a measure of the chemical affinity between the substituent and the receptor, are shown in detail in the 2D diagram in Figure 5 (left).

**TABLE 4:** Results obtained from docking analysis.

Ligand	Score	Cavity Volume
Rosmarinic acid	−8.6	6073
Coumaric acid	−7.1	6073
Luteolin	−9.7	6073
Quercetin	−9.0	6073
Eriodictyol	−9.8	6073
Hydroxybenzoic acid	−6.4	6073

A total of five main hydrogen bonds appear to form with the amino acid residues Tyr77, Tyr102(A), Tyr177(A), His188(A), and Gln252(A), with distances ranging from 2.70 to 3.20 Å. At the same time, a number of hydrophobic interactions are formed between RA and amino acids that form the active site of PAFR (Trp73(A), Phe98(A), Phe174(A), Trp255(A), His248(A)), imparting stability to the binding complex.

The deep penetration of the ligand, the high binding score (Table 4), and the number of chemical interactions within the active pocket indicate high chemical affinity and, consequently, effective inhibition of PAFR and, by extension, its inflammatory mechanisms, making RA a potent antagonist of the physiological ligand (PAF).

These results of the high affinity of RA as an anantagonist of PAF for binding on PAF‐R, come in accordance with the in vitro above‐mentioned findings of the strong anti‐PAF effects of RA (Table 2), the inhibitory effect of which against platelet aggregation was aligned with higher specificity for the PAF/PAF‐R related inflammatory pathway of the activation of platelets, which was also comparable to other well‐established anti‐PAF natural compounds, like other phenolics [18], polar lipids [29], and drugs [12, 13].

#### 2.6.2 Docking of CA With PAFR

CA is a plant metabolite with anti‐inflammatory and antioxidant properties, while ex vivo and in vitro studies have also demonstrated its antiplatelet effects through the reduction of thromboxane production. In addition, it appears to be involved in the AA metabolic pathway, reducing the production of thromboxane B2 and prostaglandin E2, which are induced by lipopolysaccharides [42].

CA showed moderate binding to PAFR, as indicated by the binding score of −7.1 kcal/mol according to blind docking in AutoDock Vina (Table 4). The active compound appears to be located in the same binding pocket (C1) of PAFR, with a cavity volume (6073 Å^3^). However, CA appears to occupy only a small part of this cavity. The insufficient occupation of the site is consistent with the limited number of polar and nonpolar interactions involved in the formation and stabilization of the binding complex.

**FIGURE 6:**
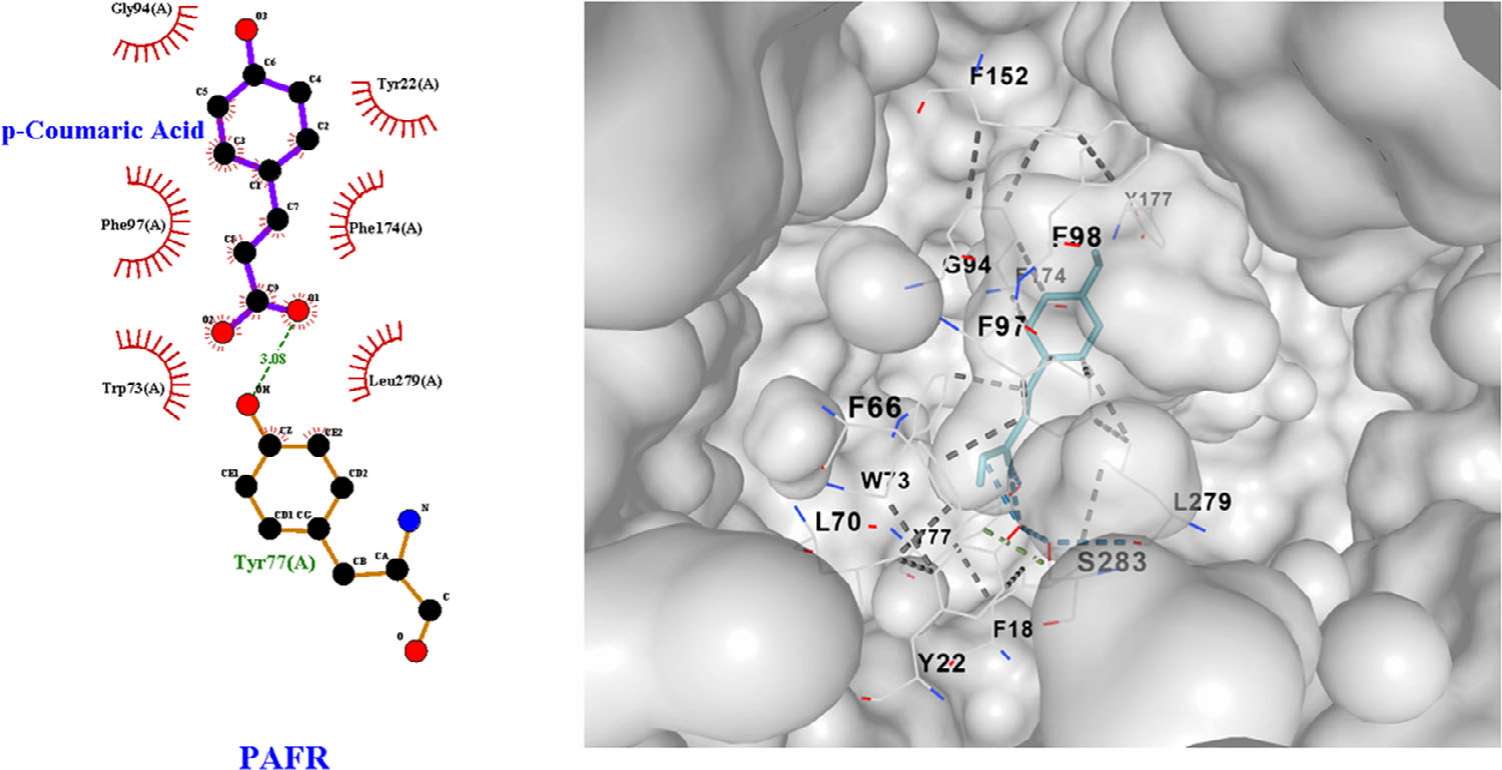
Two‐dimensional interaction diagram (left) and 3D binding pose (right) of coumaric acid (CA) docked to the PAF receptor. A Hydrogen bond between CA and PAFR highlighted in the 2D diagram and residues Tyr77(A) (green labels), as well as hydrophobic interactions with Gly94(A), Phe97(A), Leu279(A) (red arcs). The positioning of CA within the PAFR binding pocket and the resulting key interactions with amino acid residues are shown in the 3D structure.

As shown in detail in the 2D diagram in Figure 6 (left), one hydrogen bond appears to be formed with the amino acid Tyr77(A), with a bond distance of 3.08 Å. Also, a small number of hydrophobic interactions are formed between CA and amino acids involved in the formation of the active site of PAFR (Gly94(A), Phe97(A), Leu279(A)), but not as many as the number of interactions in RA, which makes the binding weaker. The 3D diagram (Figure 6, right) shows that CA binds near aromatic residues, without significant involvement in π‐π stacking, which further justifies the moderate binding affinity.

These results on the lower but considerable affinity of p‐CA for the antagonism of PAF on PAF‐R, come in accordance with the in vitro above‐mentioned findings of the less potent anti‐PAF effects of CA compared to that of the RA (Table 2). Nevertheless, as shown in Table 2, even though the p‐CA showed less potent anti‐PAF effect, still this inhibitory effect against platelet aggregation was also comparable to some extend with other well‐established anti‐PAF natural compounds, like other phenolics [18], polar lipids [29] and drugs [12, 13]. This suggests, that even though p‐CA is less potent compared to RA against PAF, still its copresence in the extract further enhance the overall anti‐PAF and anti‐ADP activities of the R. *rubiginosa* extract, highlighting the functional anti‐inflammatory and antithrombotic potential for this extract

#### 2.6.3 Docking of Luteolin With PAFR

Although the mechanisms of luteolin's antiplatelet activity are not yet clear, it has been found to inhibit platelet aggregation induced by convulxin and collagen and inhibit platelet ROS production, increasing their antioxidant capacity, according to a study by Yujia Ye et al. [43].

**FIGURE 7:**
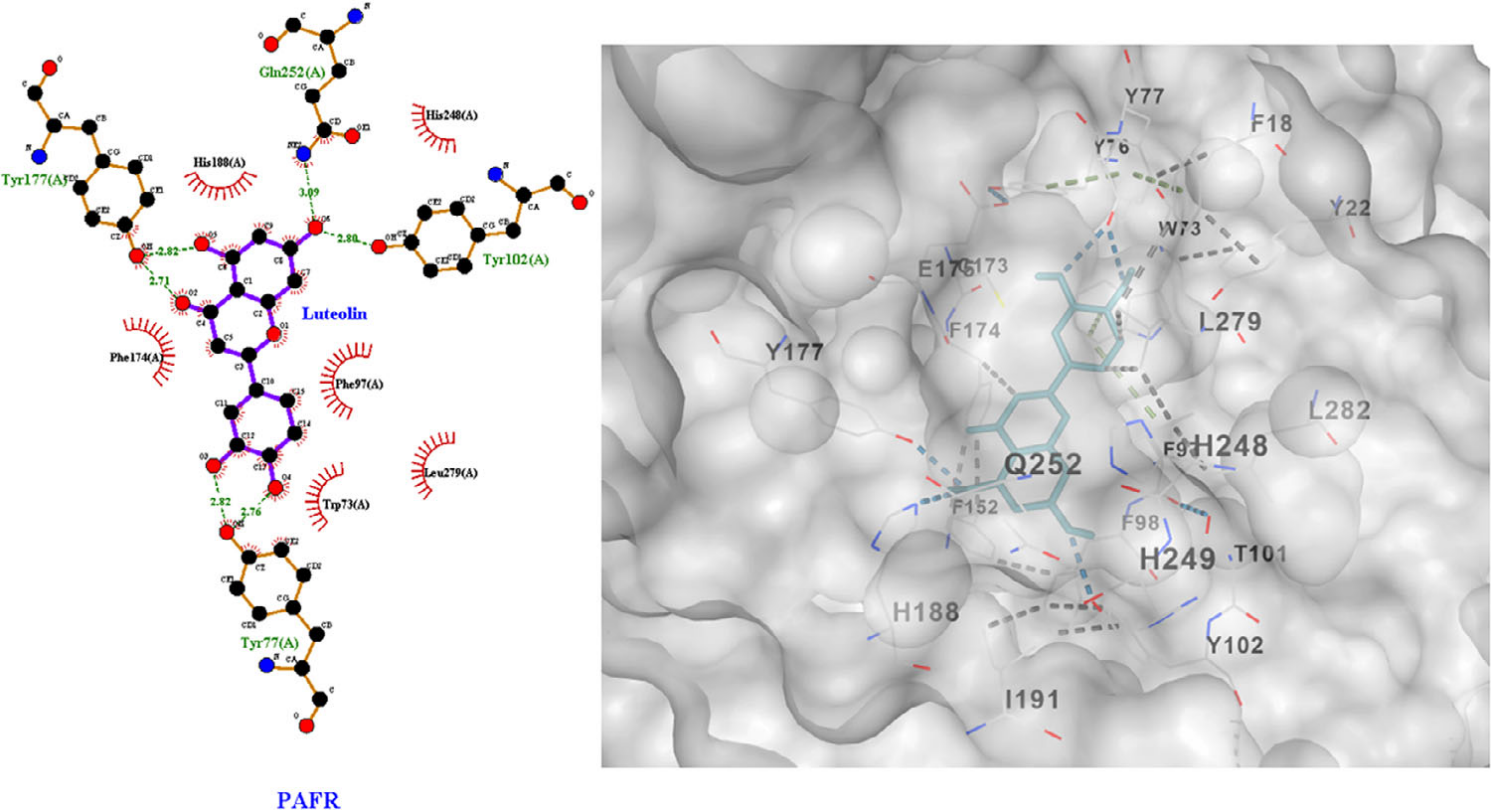
Two‐dimensional interaction diagram (left) and 3D binding pose (right) luteonil docked to the PAF receptor. Hydrogen bonds between luteonil and PAFR highlighted in the 2D diagram and residues Tyr77(A), Tyr102(A), Tyr177(A), and Gln252(A) (green labels), as well as hydrophobic interactions with Trp73(A), Phe174(A), His188(A) (red arcs). The positioning of luteonil within the PAFR binding pocket and the resulting key interactions with amino acid residues are shown in the 3D structure.

Luteolin yielded a binding score of −9.7 kcal mol^−1^ according to blind docking (Table 4). The active compound appears to be located in the binding pocket C1 of PAFR, with a cavity volume (6073 Å^3^), occupying a large volume of the cavity and forming both polar and nonpolar interactions (Figure 7).

These interactions, are shown in detail in the 2D diagram in Figure 7 (left). Six main hydrogen bonds appear to form with the amino acid residues Tyr77(A), Tyr102(A), Tyr177(A), and Gln252(A), with distances ranging from 2.71 to 3.09 Å. Also a number of hydrophobic interactions are formed between Luteolin and amino acids that form the active site of PAFR (Trp73(A), Phe174(A), His188(A)), imparting stability to the binding complex.

#### 2.6.4 Docking of Quercetin With PAFR

In vitro test results have shown that flavonoids have high antiplatelet activity, particularly a combination of catechin and quercetin, making quercetin a key compound in enhancing antiplatelet action due to its ability to engage in PAF‐related signaling mechanisms for platelet activation by binding to PAFR [18].

**FIGURE 8:**
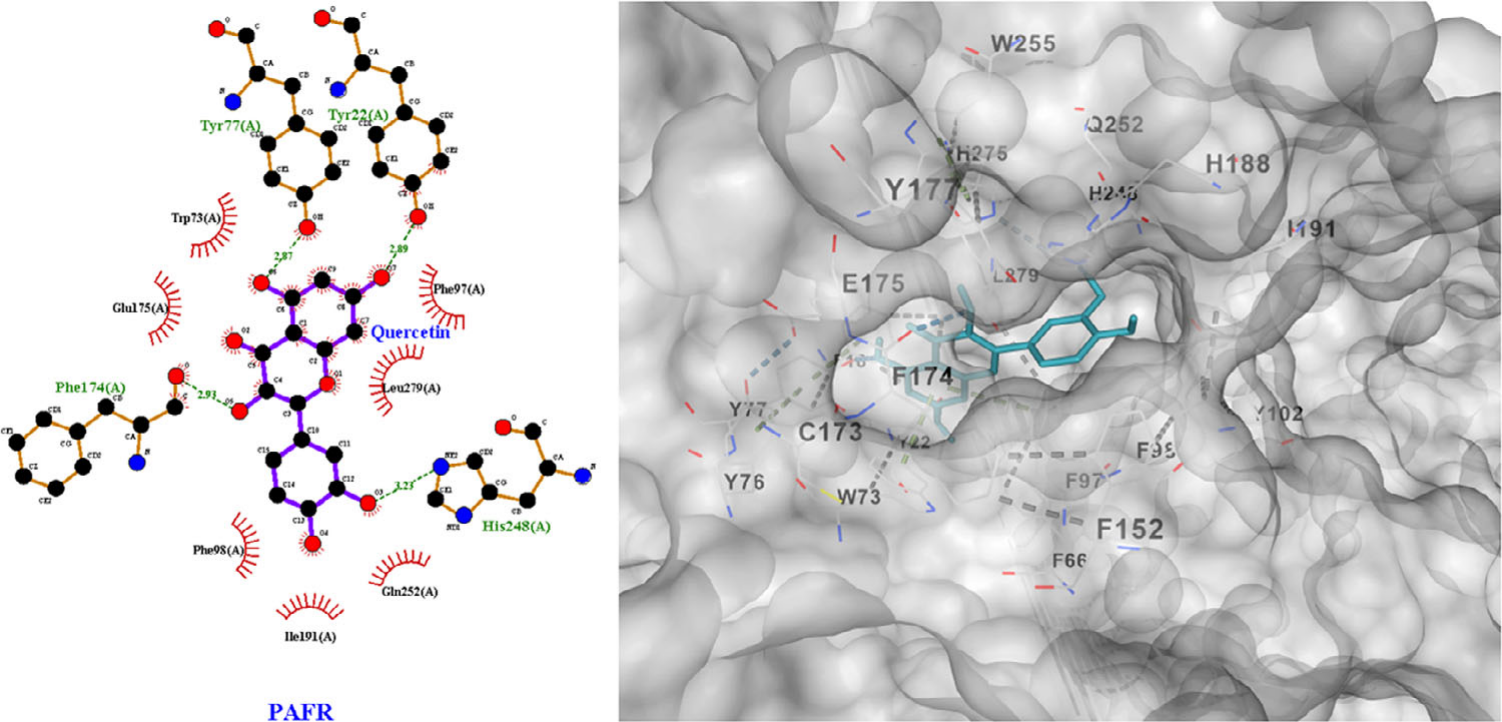
Two‐dimensional interaction diagram (left) and 3D binding pose (right) of quercetin docked to the PAF receptor. Hydrogen bonds between quercetin and PAFR highlighted in the 2D diagram and residues Tyr22(A), Tyr77(A), Phe174(A), His248(A) (green labels), as well as hydrophobic interactions with Phe97(A), Phe98(A), Gln252(A) (red arcs). The positioning of quercetin within the PAFR binding pocket and the resulting key interactions with amino acid residues are shown in the 3D structure.

Quercetin was used in docking. It targeted the same active site as the other ligands (6073 Å^3^ cavity volume) and produced the optimum score of −9.0 kcal mol^−1^ (Table 4). The interactions of quercetin with PAFR and the hydrogen bonds are shown in detail in the 2D diagram in the Figure 8 (left). Hydrogen bonds between quercetin and PAFR are formed with the amino acids Tyr22(A), Tyr77(A), Phe174(A), His248(A). A combination of hydrophobic interactions and hydrogen bonds explains quercetin's near‐top ranking, confirming our review's observation that polyphenols are strongly associated with immune targets.

These results of the in silico derived potential high affinity of quercetin as an anantagonist of PAF for binding on PAF‐R, come in accordance with the above‐mentioned in vitro findings of the strong anti‐PAF effects of quercetin (Table 2), the inhibitory effect of which against platelet aggregation was also comparable to other well‐established anti‐PAF natural compounds, like other phenolics [18], polar lipids [29], and drugs [12, 13].

#### 2.6.5 Docking of Eriodictyol With PAFR

In vitro studies have reported Eriodictyol ability to inhibit platelet aggregation, which is caused by thrombin and moderating ROS production in activated platelets. Eriodictyol exhibiting antiplatelet activity with IC_50_, which is an additional finding for the analysis of its molecular binding to PAFR [44, 45].

**FIGURE 9:**
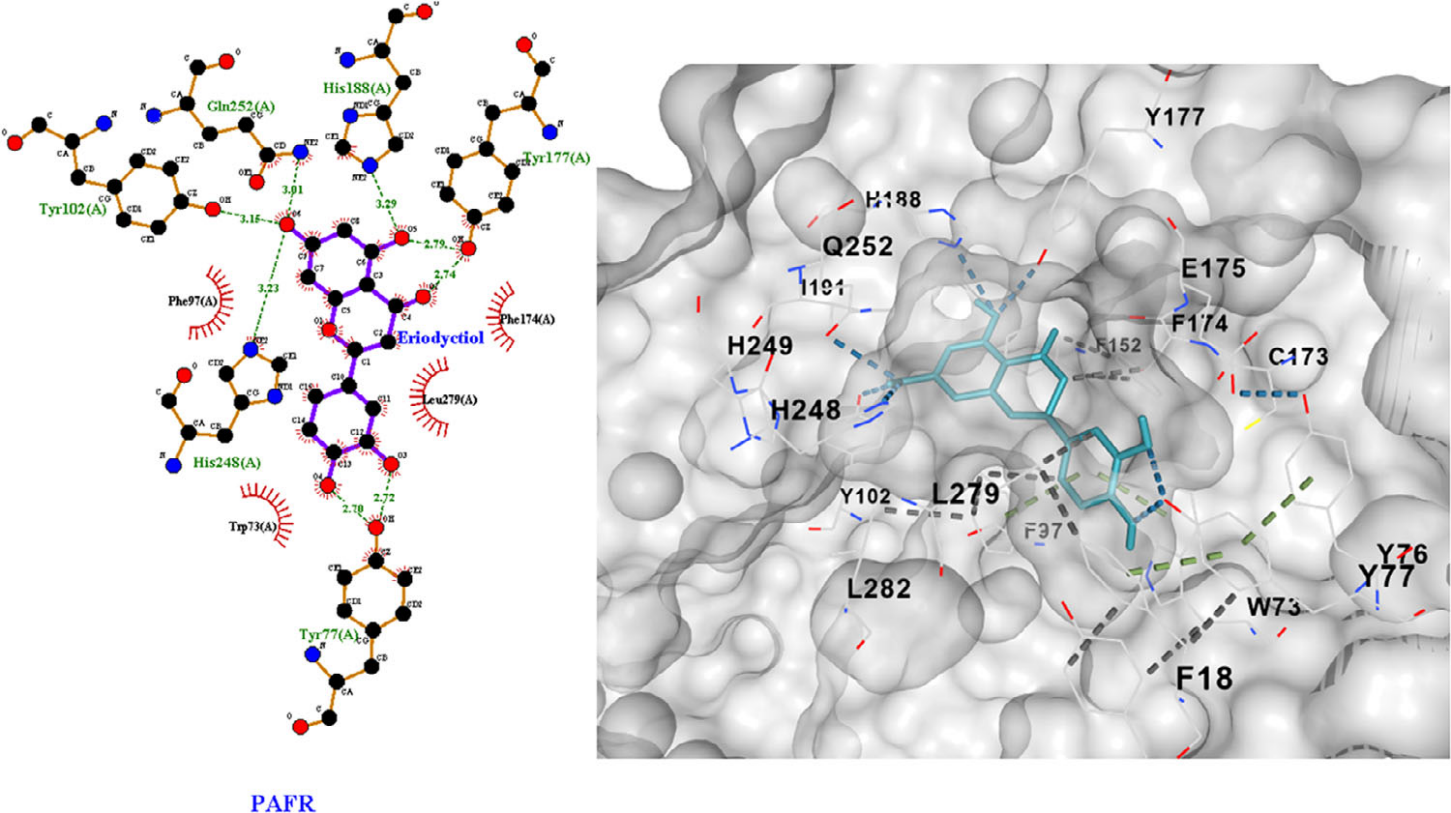
Two‐dimensional interaction diagram (left) and 3D binding pose (right) of eriodyctiol docked to the PAFR. Hydrogen bonds between Eriodyctiol and PAFR highlighted in the 2D diagram and residues Tyr77(A), Tyr102(A), Tyr177(A), His188(A), His248(A), and Gln252(A) (green labels), as well as hydrophobic interactions with Phe97(A) and Phe174(A) (red arcs). The positioning of eriodyctiol within the PAFR binding pocket and the resulting key interactions with amino acid residues are shown in the 3D structure.

Eriodictyol appears to exhibit deep binding in the C1 pocket of PAFR, yielding the strongest binding score, a total of −9.8 kcal/mol (Table 4), which indicates high chemical affinity. When Eriodictyol binds to PAFR, it appears that the polyphenyl ring is anchored to the pocket by forming multiple strong hydrogen bonds with certain key amino acid residues, Tyr77(A), Tyr102(A), Tyr177(A), His188(A), His248(A), Gln252(A), and bond lengths ranging from 2.70–3.23 Å, which provide stability and determine the orientation of the compound at a point. However, orientation and stability are also controlled and enhanced by a set of hydrophobic interactions with amino acids Phe97(A) and Phe174(A). All interactions are visible in detail in the 2D diagram in Figure 9. The 3D diagram highlights the centered deep placement of the eriodyctiol skeleton in PAFR and the large volume it occupies in the active site.

#### 2.6.6 Docking of HΒA With PAFR

HΒA and its derivatives also exhibit antithrombotic properties, inhibiting the formation of fibrin clots and providing an additional antithrombotic protective mechanism [46].

The molecular docking analysis of HΒA, compared to polyphenolic compounds, yielded low affinity and weak binding to the active site of PAFR (C1), with a binding score of −6.4 kcal/mol, as expected due to its small size and limited ability to form interactions during binding (Table 4).

**FIGURE 10:**
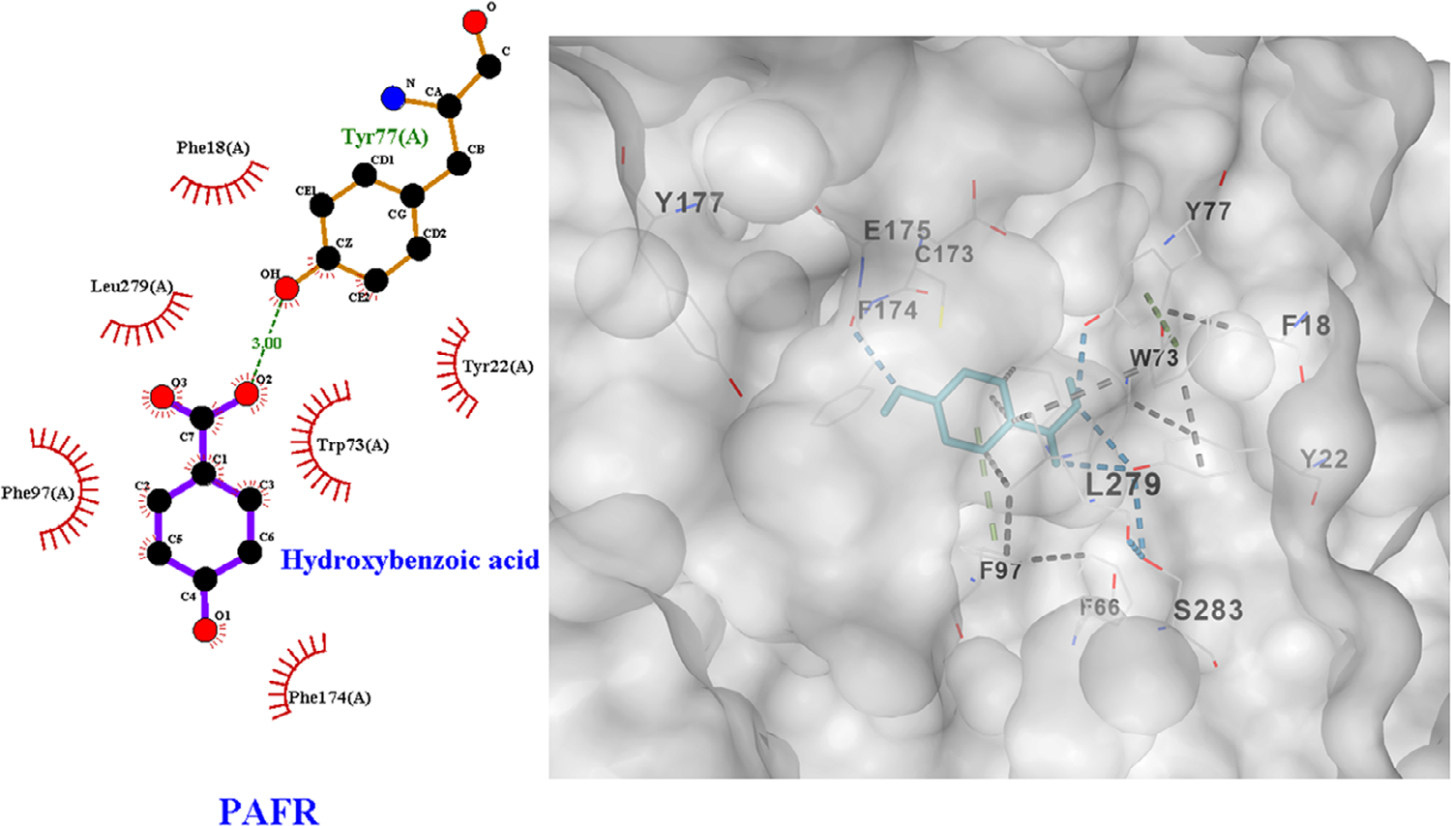
Two‐dimensional interaction diagram (left) and 3D binding pose (right) of hydroxybenzoic acid docked to the PAF receptor. A Hydrogen bond between HBA and PAFR highlighted in the 2D diagram and residues Tyr77(A) (green labels), as well as hydrophobic interactions with Phe18(A), Phe97(A), and Phe174(A) (red arcs). The positioning of HBA within the PAFR binding pocket and the resulting key interactions with amino acid residues are shown in the 3D structure.

As shown in the 2D diagram (Figure 10 left), during binding, a clear hydrogen bond is formed with Tyr77(A), at a distance of 3.00 Å, and hydrophobic contacts with amino acids such as Phe18(A), Phe97(A), and Phe174(A). The combination of the limited number of interactions and the inadequate positioning during binding explain the lower energy benefit compared to polyphenolic compounds.

### 2.7 Antimicrobial Activity of *R. rubiginosa* Extract

The antimicrobial activity of *R. rubiginosa* extract was evaluated against both Gram positive and Gram negative bacterial strains, including *Staphylococcus aureus*, *Listeria monocytogenes*, *Salmonella enterica*, and *Escherichia coli*. The evaluation included measuring the minimum inhibitory concentration (MIC) after incubation periods of 24 h and 7 days.

After 24 h of incubation with the extract, no inhibitory activity was detected against the Gram positive bacteria, *S. aureus* and *L. monocytogenes*, indicating that the extract had limited initial effectiveness against this group (Figure 2A,B). Unlike the other strains, *S. enterica* and *E. coli* were inhibited at a minimum concentration of 50 mg/mL (Figure 2C,D). This outcome indicates a comparatively quicker antimicrobial response of the extract toward Gram‐negative bacteria, due to its abundance of polyphenolic compounds.

**FIGURE 11:**
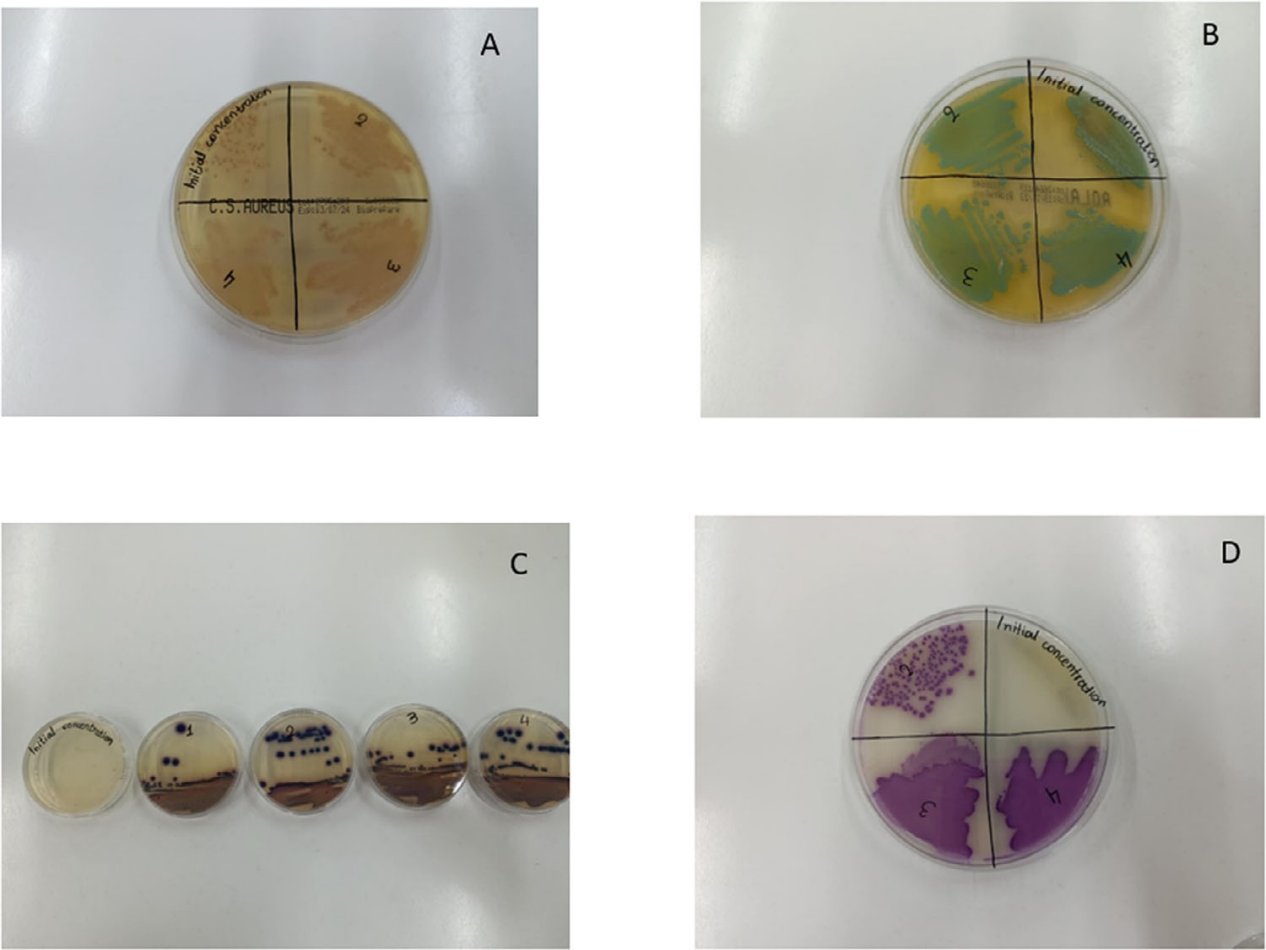
Antimicrobial action of *R rubiginosa* extract against: (A) *S. aureus*, (B) *L. monocytogenes*, (C) *E. coli*, and (D) *S. enterica*.

Notably, after 7 days of incubation, the extract's antimicrobial action broadened to include Gram positive bacteria that were initially unaffected. Both *S. aureus* and *L. monocytogenes* demonstrated susceptibility to the tested extract, with MIC value of 25 and 12.5 mg/mL, respectively (Figure 11). This delayed antimicrobial effect may indicate a time‐dependent mode of action or the gradual diffusion and accumulation of bioactive molecules within the medium [47].

In contrast to our observed moderate, time‑dependent antimicrobial activity—with better early efficacy against Gram‐negative bacteria—studies on related species such as *R. canina* or *R. damascena* generally report weak or absent antimicrobial effects under comparable phenolic‐positive extracts [48, 49].

This indicates that our extract formulation or concentration might enable more sustained antimicrobial effects. Although further research is needed to elucidate this plant extract's mechanism of action and bioactive constituents, these findings highlight its potential as an antimicrobial agent

## 3 Conclusions

This study demonstrates that *R. rubiginosa L*. extract is a rich source of phenolics, carotenoids, and other antioxidant and anti‐inflammatory bioactives exhibiting multifunctional in vitro synergistic bioactivities, including anti‐PAF, antioxidant, UV‐absorbing, and antimicrobial effects.

The whole extract showed selective inhibition of the PAF pathway in human platelets, supported by molecular docking analysis indicating favorable binding of major phenolics present in the extract to the PAF receptor, particularly eriodyctiol, luteolin, quercetin, and RA, while coumaric and HΒAs displayed weaker interactions.

These findings suggest that the phytochemical constituents of *R. rubiginosa*, especially quercetin and RA, may contribute synergistically to its anti‐inflammatory, antioxidant, and antiplatelet properties through modulation of PAF‐related mechanisms.

Overall, the results highlight *R. rubiginosa* as a promising source of bioactive compounds for functional food, nutraceutical, and nutricosmetic‐cosmetic applications.

Nevertheless, Further in vivo and clinical studies are required to confirm optimal utilization, efficacy, and safety.

## 4 Experimental Section

### 4.1 Materials, Reagents, Instrumentation, and Extract Preparation

All reagents used in the study, including Folin–Ciocalteu reagent, 1,1‐diphenyl‐2‐picrylhydrazyl (DPPH), 2,2′‐azinobis‐(3‐ethylbenzothiazoline‐6‐sulfonic acid) (ABTS), and solvents such as physiological saline—along with several standards (i.e., phenolics, Trolox, β‐carotene, etc.), were obtained from Sigma–Aldrich (St. Louis, MO, USA).

UV–vis spectroscopic analyses were conducted using an LLG‐uniSPEC 2 spectrophotometer, while for antiplatelet assays, all plastic consumables, reagents, and solvents were of analytical grade and purchased from Sigma‐Aldrich. Blood sampling was carried out using 20‐gauge safety needles and evacuated sodium citrate S‐monovettes acquired from Sarstedt Ltd. (Wexford, Ireland).

Bioassays on human platelet‐rich plasma (hPRP) were performed using a quadruple‐channel strobilometric platelet aggregometer, Chrono‐log 490 (Havertown, PA, USA), equipped with the AGGRO/LINK software package. All consumables for platelet aggregometry were sourced from Chrono‐log (Havertown, PA, USA). Standards of PAF, adenosine diphosphate (ADP), and bovine serum albumin (BSA) were also purchased from Sigma–Aldrich. Centrifugation procedures were conducted using a Nahita Blue Medimas centrifuge with a maximum speed of 4000 rpm.

The phytochemical profile was performed using a high‐performance liquid chromatography coupled with diode array detection (HPLC‐DAD). The analysis was performed using a VWR Hitachi Elite LaChrom HPLC system (VWR, Darmstadt, Germany), consisting of an autosampler (L‐2200), binary pump (L‐2130), column oven (L‐2300), and diode array detector (L‐2455). Separation was achieved on an SVEA C18 reversed‐phase column (150 mm × 4.6 mm, 5 µm particle size; Nanologica, Stockholm, Sweden) maintained at 30°C, with a constant flow rate of 0.5 mL/min. Additionally, the antimicrobial activity of the extract was evaluated in vitro against the ATCC bacterial strains.

The plant material used in this study was identified as *R. rubiginosa L*. (Rosaceae) by Dr. Evangelos Beris, Agriculturist, specialized in Plant Production, from the University of West Attica in Greece. The plant was collected from Heraklion in Crete on 17 of September. A detailed preparation of the *R. rubiginosa* extract is described in our previous work [14]. Briefly, a total of 10 g of dried, powdered plant material was macerated in a 100 mL Erlenmeyer flask and the extraction was performed using a solvent mixture of 90% distilled water and 10% ethanol—a safe, economical, and environmentally friendly system known for effectively preserving phytochemicals. To preserve the integrity and activity of the phytochemicals, extracts were stored under conditions that minimize degradation. These included refrigeration at 2°C to protect heat‐sensitive compounds, storage in the dark to avoid light‐induced oxidative degradation, and use of airtight containers to limit oxidation [50].

### 4.2 TCC Analysis

The TCC of each extract was quantified based on the method as described by Tsoupras et al. [29]. Each sample was dissolved in 2 mL of octane, and absorbance was measured at 450 nm. Carotenoid concentration was calculated using a β‐carotene standard curve, with results expressed in mg β‐carotene equivalents (CE) per gram of DW of each extract.

### 4.3 Assessment of Antioxidant Activities of Supplements

To assess the antioxidant activity of the extracts, three bioassays were employed: the DPPH radical scavenging assay, the ABTS radical cation decolorization method, and the ferric‐reducing antioxidant power (FRAP) assay, following the protocol described in Tsoupras et al. [29].

#### 4.3.1 DPPH Assay

For each extract, 0.2 mL of ethanol was mixed with 0.8 mL deionized water and 1 mL of DPPH solution. Samples were vortexed after each reagent addition and incubated in the dark at room temperature for 30 min. Absorbance was then measured at 517 nm. A blank solution containing 0.2 mL ethanol, 0.8 mL water, and 1 mL DPPH solution was prepared and measured under the same conditions. The percentage of DPPH radical inhibition was calculated as follows:

$$\text{Inhibition}\left(\%\right)=\left\{\left(A_{1}-A_{2}\right)*100\right\}/A_{1}w$$

where *A*_1_ is the absorbance of our sample solution and *A*_2_ the absorbance of the test sample solution. Afterward, IC_50_ (the concentration of each sample which is able to neutralize 50% of DPPH radicals, was calculated and the DPPH radical scavenging activity of the sample was expressed as TEAC using the following equation:

$$TEAC=IC_{50}\;of\;Trolox\left(\mu g/L\right)/IC_{50}\;of\;sample\left(\mu g/L\right)$$

#### 4.3.2 ABTS Assay

For the ABTS assay, 2 mL of ABTS reagent was added to the extract, vortexed, and incubated in the dark for 7 min. Absorbance was measured at 734 nm, with deionized water used as the blank. Trolox was used as the standard, with absorbance values ranging from 0.2 to 0.8 to construct the standard curve. Results were expressed as µmol Trolox equivalents per gram dry weight (µmol TE/g DW) calculated by:

$$ABTS\left(\mu mol\;TE/GDW\right)=C*V*t/m$$

where *C* represents the Trolox concentration obtained from the standard curve of the diluted sample, *V* is the volume of the sample, *t* is the diluting factor, and *m* the weight of the sample.

#### 4.3.3 FRAP Assay

For the FRAP assay, 3 mL of FRAP reagent was added to each dried extract, vortexed, and incubated in the dark at room temperature for 15 min. Absorbance was measured at 593 nm. Trolox was again used as a standard with concentrations ranging from 0.2 to 0.8. Results were expressed as µmol TE/g DW using the same formula as the ABTS assay.

All antioxidant assays included generation of dispersion graphs to illustrate the relationship between concentration and absorbance for each sample.

### 4.4 Assessment of Anti‐UV Photoprotective and Antiaging—Anti‐inflammatory Potential of Rose Extracts

The anti‐UV photoprotective potential of Rose extracts was measured by conducting a total UV–vis spectra analysis of the sample, which was dissolved in 2 mL of ethanol for this analysis, in order to acquire evidence of the present of compounds in the extract that are able to absorb UV–radiation, along with its SPF evaluation, as previously described [28]. Before the analysis the absorbance of the ethanol solvent was set as blank. Moreover, the estimation of the SPF was performed by UV–vis spectroscopy, recording the absorption of the samples at wavelengths from 290 to 320 nm, every 5 nm. Measurements were performed on a UV–vis spectrophotometer using 1 cm quartz cells, with ethanol used as a blank sample. SPF was calculated using the Mansur equation:

$$SPF=CF\times \sum EE\left(\lambda \times I\right)\left(\lambda \right)\times ABS\left(\lambda \right),$$

where CF is a correction factor (= 10), EE(*λ*) is the spectrum of erythematogenic effect, that is, the effectiveness of UVB radiation in causing redness and inflammatory reaction in the skin; *I*(*λ*) is the intensity spectrum of solar radiation; and Abs(*λ*) is the absorbance of the cream at the respective wavelengths. The constant values of the coefficients are shown in Table 5.

**TABLE 5:** Parameters used to calculate the SPF.

Wavelength *λ* (nm)	EE (*λ*) × *I* (*λ*)^a^
290	0.015
295	0.082
300	0.287
305	0.328
310	0.186
315	0.084
320	0.018

^a^ EE (*λ*): erythematogenic action spectrum, *I*(*λ*): solar irradiance spectrum.

The antiaging—anti‐inflammatory potential of the tested extracts were evaluated by quantifying the anti‐PAF efficacy of the extract versus standard compounds in a well‐established biological system to assess these kind of studies by using hPRP obtained from healthy donors (*N* = 6). The anti‐inflammatory inhibitory effect of the extract on platelet aggregation was assessed following stimulation of platelets with PAF in the presence of the test compounds, compared to tests in the absence of the compounds as described in Tsoupras et al. [13, 29]. Similar experiments were conducted for the tested compounds against a classic platelet agonist in order to evaluate the antiplatelet antithrombotic efficacy of the tested samples against the classic ADP‐pathway of platelet aggregation. Results are expressed as mean IC_50_ values ± standard deviation, representing the concentration/amount (µg) of the bioactive extract required to achieve 50% inhibition of PAF/ADP‐induced platelet aggregation. Each sample was tested multiple times using blood samples from different healthy donors to ensure reproducibility and reliability.

Ethics Statement: In this study, the evaluation of the anti‐inflammatory and antithrombotic properties of such natural bioactives was performed in vitro in human PRP obtained from blood samples of healthy donors/volunteers, which were conducted in accordance with the Declaration of Helsinki and approved by the Ethics Committee of Democritus University of Thrace (protocol code ΔΠΘ/ΕHΔΕ/7690/70, 27 September 2024).

Informed Consent Statement: “Informed consent was obtained from all healthy blood donors involved in the study”.

### 4.5 Analytical Conditions—HPLC‐DAD Analysis

The analytical method was based on the protocol described by Kouri et al. [51] with minor modifications. The mobile phase consisted of solvent A (water with 1% formic acid), solvent B (methanol with 1% formic acid), and solvent C (acetonitrile with 1% formic acid). The gradient elution was programmed as follows: 0–5 min: 90% A, 6% B, 4% C, 5–30 min: 85% A, 9% B, 6% C, 30–60 min: 71% A, 17.4% B, 11.6% C, 60–63 min: 0% A, 85% B, 15% C, 63–65 min: 90% A, 6% B, 4% C. The injection volume was 20 µL, and detection was carried out at 280 nm. All samples were analyzed in triplicate. Quantification was based on external calibration curves prepared from individual standard compounds (15 mg) dissolved in methanol (50 mL) to yield stock solutions of 300 µg/mL. Serial dilutions were prepared to obtain working solutions ranging from 1 to 100 µg/mL. Each compound was quantified using a five‐point linear calibration curve with a coefficient of determination (*R* ^2^) > 0.98. Standards were selected based on relevant literature [52, 53]. Identification of flavophotonoids and phenolic acids in the extracts was carried out by comparing the retention times and maximum absorption wavelengths of analytes with those of the reference standards, and by spiking samples with internal standards.

### 4.6 In Vitro Evaluation of Antimicrobial Activity

Bacterial cultures were prepared at a concentration of approximately 1.0 × 10^8^ CFU/mL, corresponding to 0.5 on the McFarland scale. Aqueous extracts at varying concentrations (1–800 µg/mL) were added to the bacterial suspensions, and 25 µL of each mixture was inoculated onto Chromogenic Agar plates (Bioprepare, Athens, Greece) selective for the tested microorganisms. Plates were incubated at 37°C for 24 h. Antimicrobial efficacy was assessed by quantifying bacterial growth and determining the MIC values. Control plates without plant extracts were included to confirm normal bacterial growth and validate the experimental conditions [54]

### 4.7 Molecular Docking Analysis

To investigate the efficiency of the binding of bioactive phytochemicals to the PAF receptor (PAFR), molecular docking analysis was performed for the main bioactive phenolic compounds identified in the *R. rubiginosa* extract, as previously described by Tsiapali et al. [28], with some minor modifications. Molecular docking is a useful and advanced tool in the study and discovery of active substances, as it allows for the rapid collection and prediction of interactions between selected active substances (ligands) under study and target proteins (receptors) [55]. Thus, its capabilities were utilized to examine the binding of each phenolic compound to the orthosteric pocket C1 of PAFR, due to the finding of optimal docking scores using Autodock (version 4.2.6.). The results obtained from the molecular binding process are presented in the form of binding energies (kcal/mol), which are a criterion for comparative evaluation of the affinity of the ligands with the receptor. It is generally accepted that more negative vina score values (usually −9 kcal/mol and below) indicate a stronger bond and better affinity, and are often observed in strong inhibitors [56]. An additional analysis of the interactions of each ligand with the amino acid residues at the active site of PAFR was performed by creating 2D diagrams using LigPlot+ software, which, in combination with the 3D diagrams and results, provide a comprehensive interpretation of the interaction, binding, and incorporation of the active substances to evaluate their inhibitory capacity for both anti‐inflammatory and antioxidant actions that they were found to provide.

#### 4.7.1 Selection of Receptor for Docking Analysis

The human PAFR (UniProt ID: P25105) was chosen as the receptor for the docking analysis, since it was also the target protein of the experimental analysis. The AlphaFold Protein Structure Database (PSD) provided its 3D structure as a .PDB file.

#### 4.7.2 Selection of Ligands for Docking Analysis

A number of six compounds were selected from PubChem Compound Database: RA, CA, Luteolin, Quercetin, Eriodictyol and HΒA, and their 3D structures was stored as an Spatial Data File (SDF). The details are presented in Tables 4 and 6.

**TABLE 6:** Selected phytochemical compounds with CIDs, illustration of their chemical structures and biological roles.

Ligand	CID	Structure^a^	Potential biological role
Rosmarinic acid	5281792	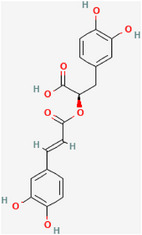	Anti‐inflammatory, antioxidant
Coumaric acid	637542	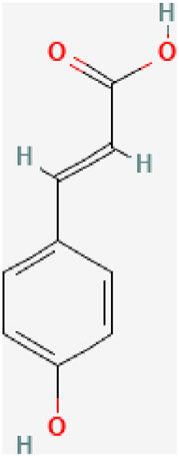	Phenolic antioxidant
Luteolin	5280445	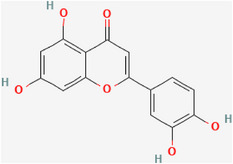	Anti‐inflammatory, antioxidant, neuroprotective
Quercetin	5280343	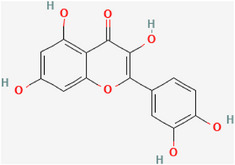	Anti‐inflammatory, antioxidant
Eriodictyol	440735	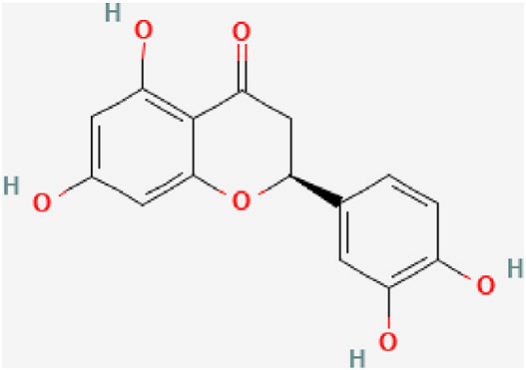	Anti‐inflammatory, antioxidant, neuroprotective
Hydroxybenzoic acid	135	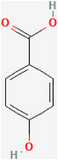	Antioxidant; constituent in food and cosmetics

^a^ Structures were obtained from Molview.com (Accessed on October 10, 2025).

#### 4.7.3 Molecular Docking of Ligands With Protein Receptor

Following the collection of the potential ligands and receptors, the CB‐Dock (Cavity‐detection guided Blind Docking) program was used to conduct three docking analyses, one for each replacement. Typically, as in this analysis, the top five clusters are chosen and examined for docking. Then, by modifying the docking box's size, the geometric center and dimensions of each cavity are determined to ensure that it is sufficiently covered without requiring extra space. Following the ligand's introduction, docking is carried out for each cavity, calculating the various binding locations (poses). These are then reranked, and the binding affinity is used to determine which is the best. For a clear representation and investigation of the interactions of the ligands during binding inside the receptor, 2D diagrams were created using the LigPlot+ program.

### 4.8 Statistical Analysis

Following data categorization, the Kolmogorov–Smirnov test was applied to assess the normality of distribution, which informed the selection of appropriate statistical analyses. A one‐way analysis of variance (ANOVA) was employed to compare IC_50_ values related to agonist‐induced platelet aggregation in human samples. For antioxidant assay comparisons, non‐parametric tests for independent samples were utilized, depending on data distribution. Statistical significance was set at *p* < 0.05. All analyses were performed using IBM SPSS Statistics for Windows, Version 29.0 (IBM Corp., Armonk, NY, USA).

## Author Contributions

**Sophia Letsiou**: conceptualization, data curation, formal analysis, investigation, writing – original draft preparation, and supervision. **Dimitrios Kranas**: formal analysis, investigation, and writing – original draft preparation. **Konstantina Kyratzi**: formal analysis, and investigation. **Aliki Tsakni**: formal analysis, investigation, and writing – original draft preparation. **Anna Ofrydopoulou**: formal analysis and investigation. **Konstantinos Pavlidis**: methodology, formal analysis, investigation, and writing – original draft preparation. **Panagiotis Halvatsiotis**: writing – review and editing. **Dimitra Houhoula**: writing – review and editing and supervision. **Alexandros Tsoupras**: conceptualization, data curation, formal analysis, investigation, methodology, writing – original draft preparation, writing – review and editing, visualization, validation, supervision, resources, and correspondence.

## Conflicts of Interest

The authors declare no conflicts of interest.

## Supporting information




**Supplementary file 1**: cbdv71418‐sup‐0001‐SuppMat.docx

## Data Availability

The data that support the findings of this study are available from the corresponding author upon reasonable request.
