# Supplementary material for: Rosa rubiginosa L. Extract Rich in Bioactive Phenolics Possess Potent Anti‐Inflammatory, Antioxidant, Photoprotective, and Antimicrobial Biofunctional Activities
Source: Chem Biodivers. 2026 Jun 15;23(6):e71418. doi: 10.1002/cbdv.71418 (PMC13268575; doi:10.1002/cbdv.71418)
Supplement: Supplementary file 1 — cbdv71418‐sup‐0001‐SuppMat.docx [file CBDV-23-e71418-s001.docx]

**Supplementary Figure 1.** Calibration Curve for Rosmarinic Acid

**Supplementary Figure 2.** Calibration Curve for *p*-Coumaric Acid

**Supplementary Figure 3.** Calibration Curve for Luteoilin

**Supplementary Figure 4.** Calibration Curve for Quercetin

**Supplementary Figure 5.** Calibration Curve for Eriodictyol

**Supplementary Figure 6.** Calibration Curve for Hydroxybenzoic Acid

**
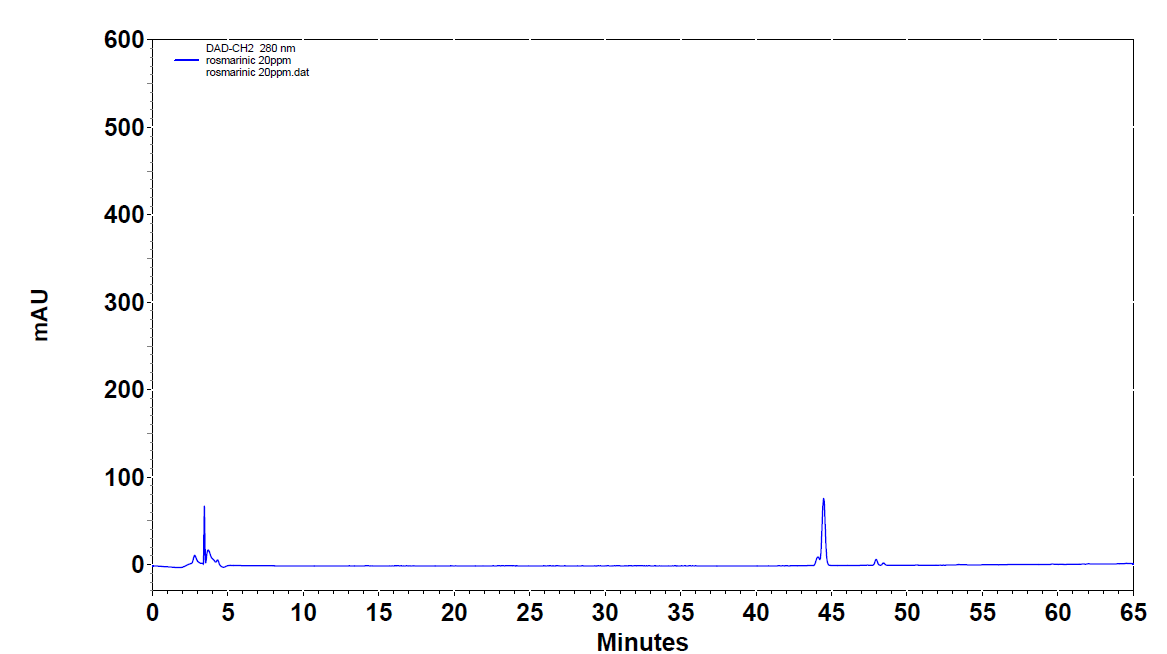
**

**Supplementary Figure 7.** Representative chromatogram for Rosmarinic Acid

**
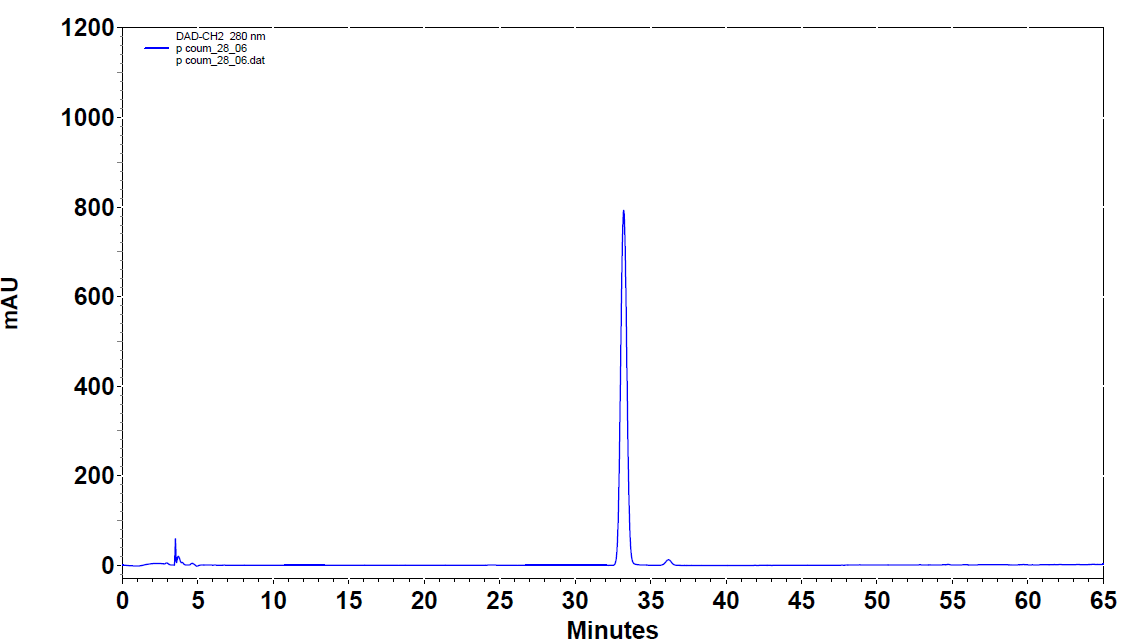
**

**Supplementary Figure 8.** Representative chromatogram for *p*-Coumaric Acid

**
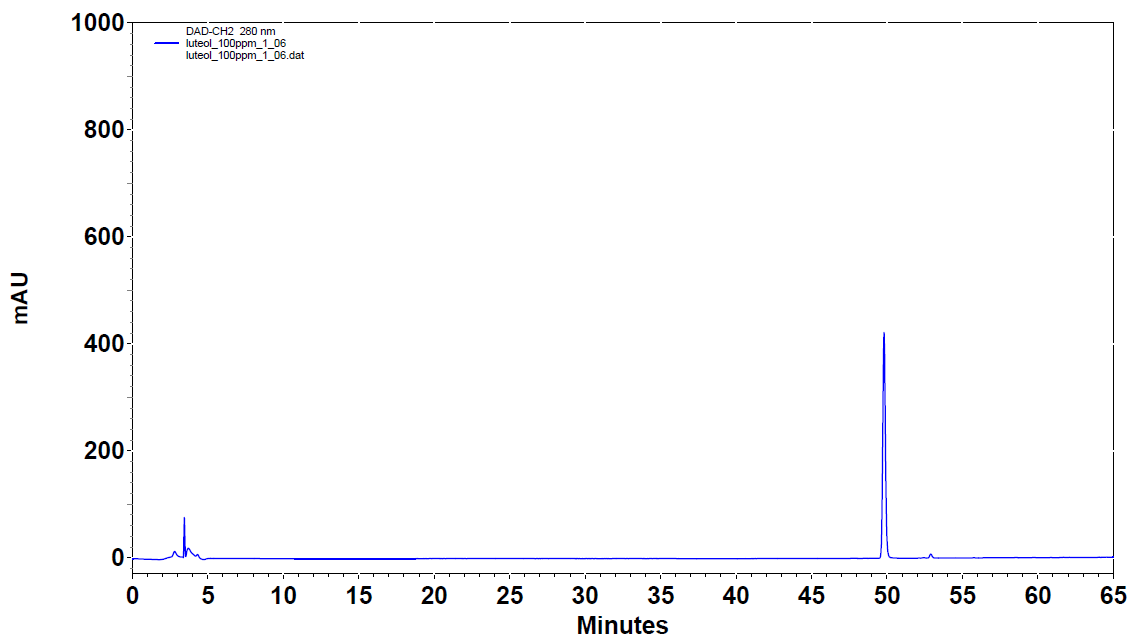
**

**Supplementary Figure 9.** Representative chromatogram for Luteolin

**
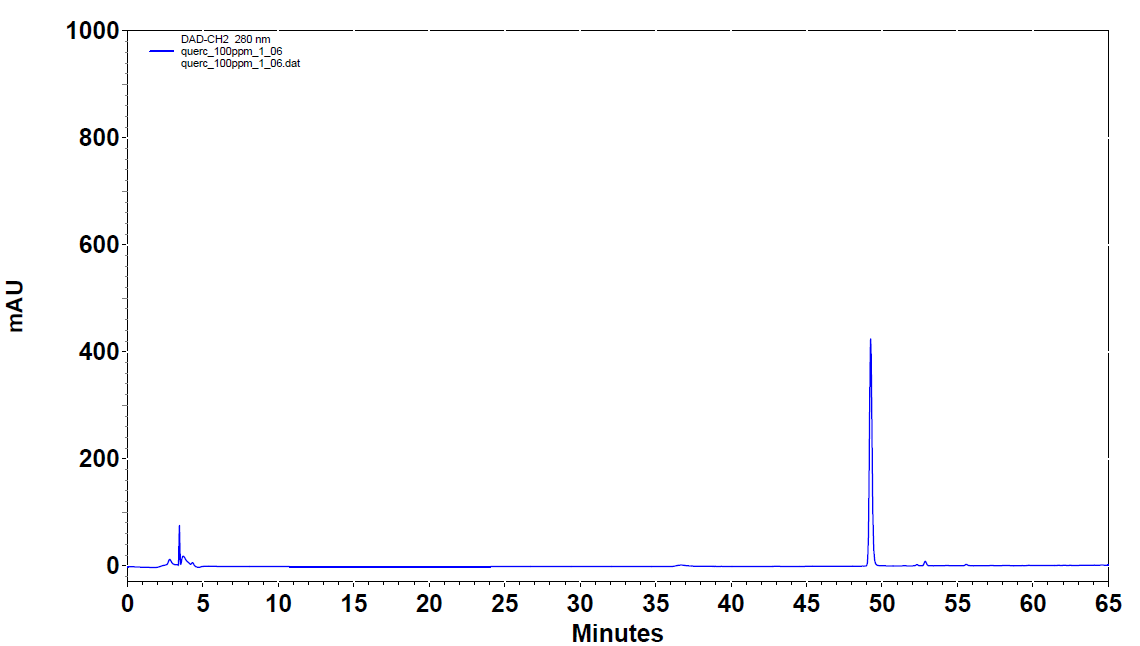
**

**Supplementary Figure 10.** Representative chromatogram for Quercetin

**
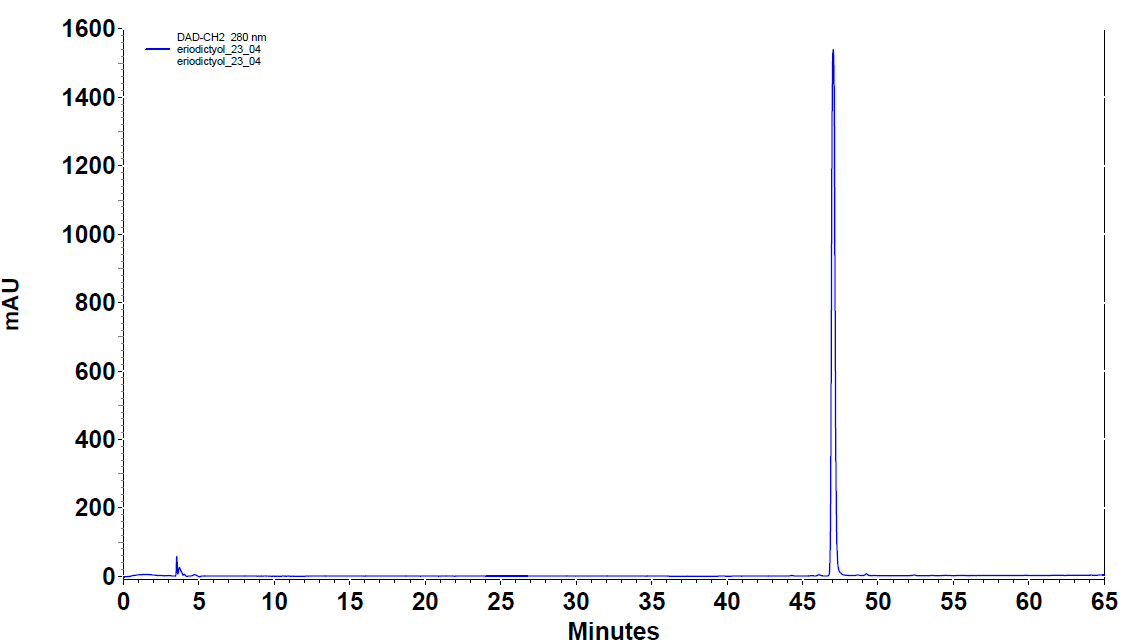
**

**Supplementary Figure 11.** Representative chromatogram for Eriodictyol

**Supplementary Table 1. Standard compounds, their retention time, calibration curves, maxima wavelengths in ultraviolet region**

|  | **Standard compounds** | **Retention time (min)** | **Calibration Curve** | **Regression Coefficient (R^2^)** | **Maxima wavelengths (nm)** |
| --- | --- | --- | --- | --- | --- |
| A. | Rosmarinic acid | 44.50 | y=342476x-1E+06 | 0.9948 | 206,245,330 |
| B. | Coumaric acid | 33.50 | y=1E+06x-268063 | 0.9962 | 216,309,395 |
| C. | Luteolin | 49.80 | y=201134x+343231 | 0.9961 | 256,263,349 |
| D. | Quercetin | 49.24 | y=208457x+441912 | 0.9992 | 230,256,370 |
| E. | Eriodictyol | 46.96 | y=462783x+901504 | 0.9993 | 205,230,288 |
| F. | Hydroxybenzoic acid | 19.29 | y=216500x-261891 | 0.9962 | 210,260,388 |
| G. | Naringenin | 50.11 | y=392749x+600702 | 0.998 | 235,289 |
| H. | Thymol | 57.99 | y=140232x+563914 | 0.9953 | 215,276,345 |
| I. | Carvacrol | 57.65 | y=96580x+159301 | 0.9987 | 221,275 |
| J. | Kaempferol | 52.03 | y=265195x-28475 | 0.998 | 250,315 |
| K. | Apigenin | 52.21 | y=407824x+599220 | 0.9992 | 267,338 |
| L. | Apigenin-7-glucoside | 43.20 | y=475911x-3E+06x | 0.9918 | 206,267,337 |
| M. | Rutin | 40.63 | y=101038x+244152 | 0.9948 | 258,356 |
| N. | Vanillic acid | 22.55 | y=280308x+1E+06 | 0.9972 | 208,265,292 |
| O. | Ferulic acid | 36.77 | y=361100x+667162 | 0.9984 | 210,322 |
| P. | Caffeic acid | 24.57 | y=460642x-243764 | 0.9934 | 209,240,323 |
| Q. | Benzoic acid | 42.23 | y=56565x+111379 | 0.9969 | 273,370 |
